# A CARP-1 functional mimetic compound is synergistic with BRAF-targeting in non-small cell lung cancers

**DOI:** 10.18632/oncotarget.25671

**Published:** 2018-07-03

**Authors:** Vino T. Cheriyan, Hashem Alsaab, Sreeja Sekhar, Jaganathan Venkatesh, Arindam Mondal, Imran Vhora, Samaresh Sau, Magesh Muthu, Lisa A. Polin, Edi Levi, Gerold Bepler, Arun K. Iyer, Mandip Singh, Arun K. Rishi

**Affiliations:** ^1^ John D. Dingell VA Medical Center, Detroit, MI, 48201, USA; ^2^ Department of Oncology, Barbara Ann Karmanos Cancer Institute, Wayne State University, School of Medicine, Detroit, MI, 48201, USA; ^3^ Department of Pathology, Wayne State University, School of Medicine, Detroit, MI, 48201, USA; ^4^ Molecular Therapeutics Program, Barbara Ann Karmanos Cancer Institute, Wayne State University, School of Medicine, Detroit, MI, 48201, USA; ^5^ Use-inspired Biomaterials and Integrated Nano Delivery (U-BiND) Systems Laboratory Department of Pharmaceutical Sciences, Eugene Applebaum College of Pharmacy and Health Sciences, Wayne State University, Detroit, MI 48201, USA; ^6^ College of Pharmacy and Pharmaceutical Sciences, Florida A&M University, Tallahassee, FL 32307, USA; ^7^ Department of Pharmaceutics and Pharmaceutical Technology, College of Pharmacy, Taif University, Taif 26571, Saudi Arabia; ^8^ Present Address: Department of Molecular Biology, Umeå University, Umeå 90187, Sweden

**Keywords:** NSCLC, TKIs, CFM, CARP-1/CCAR1, B-Raf-inhibitors

## Abstract

Non-small cell lung cancers (NSCLC) account for 85% of all lung cancers, and the epidermal growth factor receptor (EGFR) is highly expressed or activated in many NSCLC that permit use of EGFR tyrosine kinase inhibitors (TKIs) as frontline therapies. Resistance to EGFR TKIs eventually develops that necessitates development of improved and effective therapeutics. CARP-1/CCAR1 is an effector of apoptosis by Doxorubicin, Etoposide, or Gefitinib, while CARP-1 functional mimetic (CFM) compounds bind with CARP-1, and stimulate CARP-1 expression and apoptosis. To test whether CFMs would inhibit TKI-resistant NSCLCs, we first generated and characterized TKI-resistant NSCLC cells. The GI_**50**_ dose of Erlotinib for parental and Erlotinib-resistant HCC827 cells was ∼0.1 μM and ≥15 μM, respectively. While Rociletinib or Ocimertinib inhibited the parental H1975 cells with GI_**50**_ doses of ≤0.18 μM, the Ocimertinib-resistant pools of H1975 cells had a GI_50_ dose of ∼12 μM. The GI_50_ dose for Rociletinib-resistant H1975 sublines ranged from 4.5-8.0 μM. CFM-4 and its novel analog CFM-4.16 attenuated growth of the parental and TKI-resistant NSCLC cells. CFMs activated p38/JNKs, inhibited oncogenic cMet and Akt kinases, while CARP-1 depletion blocked NSCLC cell growth inhibition by CFM-4.16 or Erlotinib. CFM-4.16 was synergistic with B-Raf-targeting in NSCLC, triple-negative breast cancer, and renal cancer cells. A nano-lipid formulation (NLF) of CFM-4.16 in combination with Sorafenib elicited a superior growth inhibition of xenografted tumors derived from Rociletinib-resistant H1975 NSCLC cells in part by stimulating CARP-1 and apoptosis. These findings support therapeutic potential of CFM-4.16 together with B-Raf targeting in treatment of TKI-resistant NSCLCs.

## INTRODUCTION

Lung carcinoma is the leading cause of cancer death in not only the United States but worldwide [1–2]. With current treatments, non-small cell carcinoma (NSCLC), which accounts for approximately 85% of lung cancer cases, carries a 5 year survival of 14% for all stages [1–3]. Oncogenic epidermal growth factor receptor (EGFR) tyrosine kinase (RTK) is a driver of a significant subset of NSCLCs and is an indicator of poor prognosis. The EGFR-driven NSCLCs are unresponsive to the frontline chemotherapeutic Cisplatin. Accordingly, EGFR TKIs are widely used to treat EGFR-driven NSCLCs. The first and second generation TKIs target the enzyme activity of EGFR, and thus inhibit NSCLC cell growth and survival signaling. Interestingly, a significant subset of NSCLCs harbor classical activating mutation in the kinase domain of the EGFR [2, 4]. This deletion of EGFR exon 19 (Δ19) is associated with good clinical responses to first generation EGFR TKIs such as Gefitinib or Erlotinib [2, 4]. However, a vast majority of patients develop resistance to these TKIs due in part to activation and/or expression of alternate, redundant RTKs as well as emergence of the “gatekeeper” T790M mutation in the kinase domain of EGFR. This EGFR T790M mutation leads to resistance to most clinically available first and second generation EGFR TKIs by increasing the affinity of the receptor to adenosine triphosphate (ATP). Recently, third generation of EGFR TKIs that are non-ATP-competitive, allosteric inhibitors of mutant EGFR were developed. Following rigorous clinical testing of Rociletinib and Ocimertinib, US FDA approved Ocimertinib for clinical use. However, a number of recent pre-clinical laboratory and animal studies have investigated molecular mechanisms of emergence of NSCLC resistance to these third-generation TKIs [5, 6]. Thus, there is an urgent need to identify and devise new approaches to treat NSCLCs and their TKI-resistant phenotypes.

CARP-1 (Cell cycle and apoptosis regulator 1/CCAR1), a peri-nuclear phospho-protein, is a regulator of cell growth and apoptosis signaling [7–10]. CARP-1 has been previously shown to regulate adipogenesis by functioning as transcriptional co-activator of the steroid receptor, glucocorticoid receptor (GR). CARP-1 also regulates Adriamycin (ADR) dependent apoptosis, mediated in part through p53 co-activation. Thus, CARP-1 co-ordinates both cell growth and apoptosis, functioning as a biphasic regulator [11, 12]. CARP-1 is often overexpressed in cells experiencing stress induced by withdrawal of growth factors or chemotherapy-induced cell cycle arrest and apoptosis [7, 8, 11]. CARP-1 is also known to co-activate the E3 ligase, APC/C, which is involved in cell cycle transitions and also tumor progression. [9, 13–15]. On the basis of CARP-1 co-activation of APC/C, our recent studies further reported identification and testing of novel, small molecule inhibitors (SMIs) of CARP-1 binding with APC/C subunit APC2 [9]. These compounds, termed CARP-1 functional mimetics (CFMs), inhibit cell growth by inducing apoptosis in various cancer cell types [9, 10]. Genetic studies previously revealed that *C.elegans* CARP-1 homolog lst 3 functioned as an antagonist of EGFR signaling but an agonist of Notch signaling [16], while targeting of EGFR caused CARP-1 increase and apoptosis [8]. We have previously observed increased resistance to apoptosis induced by chemotherapeutic drugs including ADR, Etoposide, CFMs, or EGFR TKI Gefitinib in cells where CARP-1 was knocked down, implicating its critical role in growth inhibition by these agents [7, 8, 11].

Given that EGFR TKIs remain frontline therapies for a large subset of NSCLCs, and emergence of resistance to TKIs continues to be a significant and unmet challenge, we investigated (a) whether CFM compounds inhibit NSCLC cell growth and (b) the molecular mechanisms by which CFMs inhibit growth of NSCLC cells. In addition, we investigated whether CFMs will also inhibit growth of TKI-resistant NSCLC cells. To this end, we first generated and characterized laboratory models of NSCLC cells that harbor mutant EGFR and are resistant to Erlotinib, Rociletinib, or Ocimertinib. Our studies revealed that CFM compound 4.16 inhibited growth of parental and also the TKI-resistant NSCLC cells when used as a single agent. CFM-4.16 synergized with B-Raf-targeting therapies (Sorafenib or Dabrafenib) *in vitro*. Interestingly. we also observed superior inhibition of Rociletinib-resistant NSCLC cell derived tumor xenografts in immunocompromised mice when treated with a combination of CFM-4.16 nano-lipid formulation and Sorafenib.

## RESULTS

### CFM compounds inhibit growth of NSCLC cells

**Figure 1:**
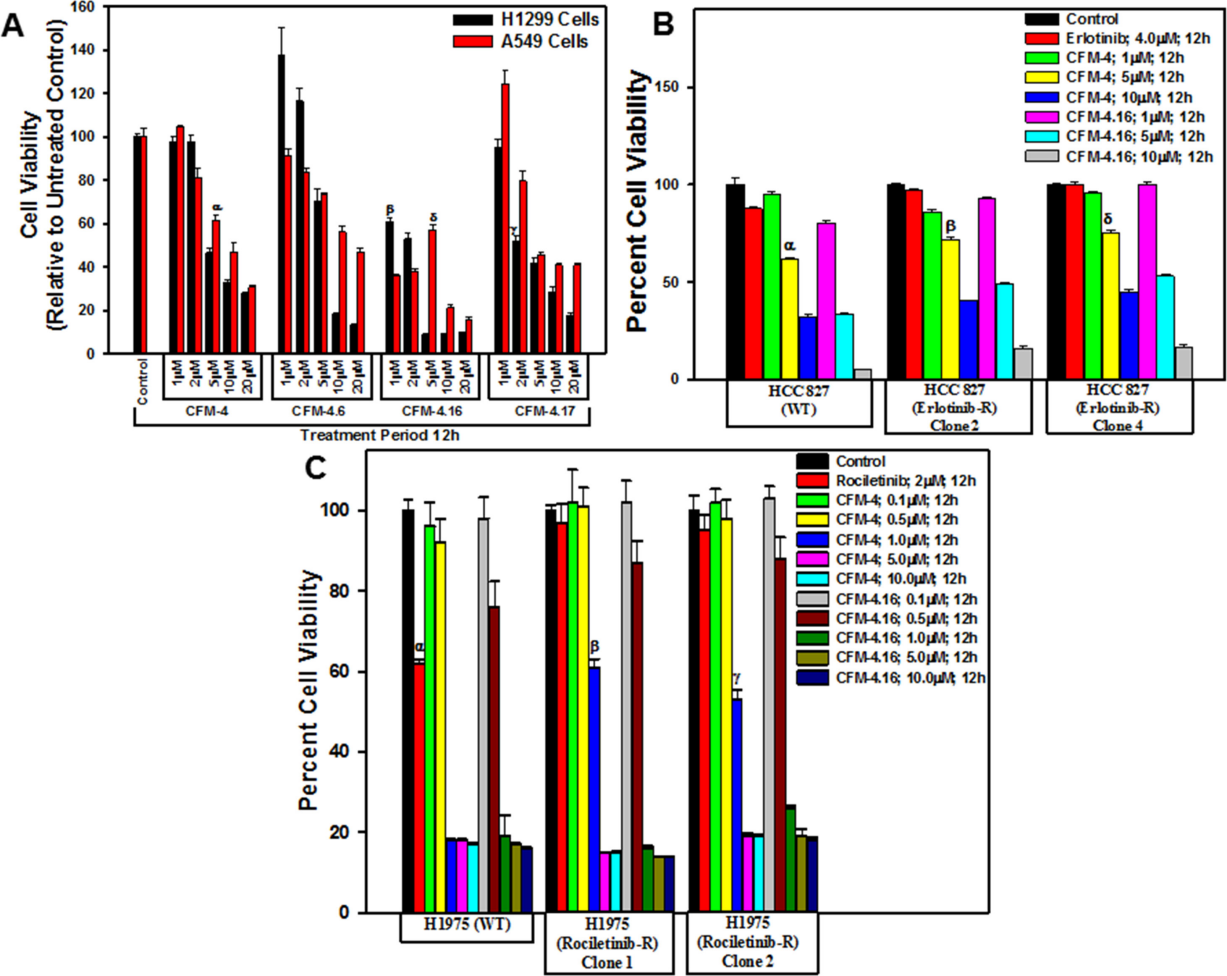
CFMs inhibit NSCLC cell growth Noted cell lines were either treated with DMSO (Control), with various CFMs (**A**–**C**), Erlotinib (B), or Rociletinib (C) for indicated dose and time. Cell viability was determined by MTT assay. The data in the histograms represent means of three independent experiments; bars, S.E. α, β, γ, δ, *p* ≤ 0.05 relative to the respective DMSO-treated controls.

We have previously reported that CFM compounds possess anti-cancer properties [9, 17–20], and we further observed that the CFM-4.16 analog effectively inhibited the growth of parental as well as drug resistant Renal cell carcinoma (RCC), and human and murine triple negative breast cancer (TNBC) cells *in vitro* and also *in vivo*. [21, 22]. Given that development of drug-resistant NSCLCs remain a formidable problem that contributes to treatment failure and poor prognosis [2–6], we tested whether CFMs, in particular CFM-4.16, would inhibit parental and drug-resistant NSCLC cells. As a proof-of-concept study, we treated the A549 and H1299 NSCLC cells with various doses of CFM-4 and its analogs CFM-4.6, -4.16 and -4.17, and determined the viabilities of these cells by MTT assays. A 10 μM and 20 μM dose of each of the compounds caused significant loss of viability of both the NSCLC cells (Figure 1A). Although treatments with a 5 μM dose of CFM-4, -4.16, or -4.17 also resulted in reduced viabilities of NSCLC cells when compared with their respective, DMSO-treated controls, CFM-4.16 was generally more potent when compared with other three compounds (Figure 1A). Further dose response analyses with reference to A549 and H1299 NSCLC cells (Figure 1A) and other NSCLC cells with mutant EGFR (see below) revealed that GI_50_ and LC_50_ of CFM-4.16 were 2.0 μM and 5–5.8 μM respectively (not shown).

We next determined whether CFMs also inhibit growth of the EGFR TKI-resistant NSCLCs. We first developed and characterized NSCLC cells that were resistant to EGFR TKIs Erlotinib, Rociletinib, or Osimertinib by culturing them in the continual presence of the respective TKIs until resistance was observed. Since, Erlotinib is frequently used in clinic for treatment of the NSCLC tumors with activating mutation in the kinase domain of EGFR [4], we chose the HCC 827 NSCLC cells with EGFR exon 19 (Δ19) mutation for generation of the Erlotinib-resistant cells. As shown in Table 1, the GI_50_ doses of Erlotinib for parental and resistant HCC827 cells were ∼0.1 μM and ≥15 μM, respectively. With growing evidence suggesting that development of resistance the TKIs Erlotinib or Gefitinib often involves activation as well as overexpression of other RTKs such as cMet or Alk, a significant subset of resistant tumors often also acquire additional, activating mutations in EGFR kinase domain. These mutations include the L858R change as well as the “gatekeeper” T790M substitution that collectively render EGFR to become constitutively active [4]. Additional allosteric, non-ATP-competitive EGFR TKIs were recently identified and the two compounds Rociletinib and Osimertinib were tested in clinical trials with subsequent and recent FDA approval of Osimertinib for use in treatment of resistant NSCLCs. Since recent laboratory studies have reported development of resistance to Rociletinib or Osimertinib in NSCLC cells [5], we chose H1975 NSCLC cells with EGFR T790M and L858R mutations for generation of Rociletinib or Osimertinib-resistant cells. The GI_50_ doses for Rociletinib and Osimertinib for the parental H1975 cells were 0.18 and 0.17 μM, respectively. Although the pools of the Osimertinib-resistant H1975 cells had the GI_50_ dose of ∼12 μM, the GI_50_ doses of Rociletinib ranged from ∼4.5 to ∼8.0 μM for the Rociletinib-resistant H1975 sublines. Of note is the finding that the Rociletinib-resistant H1975 sublines 1 and 2 that elicited ∼8.0 and 7.5 μM of Rociletinib GI_50_ dose respectively, were also resistant to Osimertinib with the GI_50_ dose of ≥0.5 μM. The data in Table 1 Clearly indicate that all the NSCLC cells developed resistance to the respective TKIs.

**Table 1:** GI50 values of parental and TKI-resistant NSCLC cells

NSCLC cell line	Erlotinib (72 h)	
		GI_50_ (µM)
HCC 827 (EGFRΔ19)	Wild type	∼0.1
	Erlotinib-R Clones 1–5	≥15.0
H1975 (EGFR T790M plus L858R)	**Rociletinib (72 h)**	
		**GI_50_ (µM)**
	Wild type	<0.18
	Rociletinib-R Clone 1	∼8.0 [≥0.5]
	Rociletinib-R Clone 2	∼7.5 [≥0.5]
	Rociletinib-R Clone 3	∼6.5
	Rociletinib-R Clone 4	∼6.0
	Rociletinib-R Clone 5	∼4.5
H1975 (EGFR T790M plus L858R)	**Osimertinib (72 h)**	
		**GI_50_ (µM)**
	Wild type	0.17
	Osimertinib-R pools	∼12.0

The parental and erlotinib-resistant (Erlotinib-R) sublines were treated with 0.1, 0.5, 1.0, 2.0, 4.0, 6.0, 8.0, 10.0, and 15.0 µM dose of Erlotinib for 72 h. In the case Rociletinib-resistant cells, the respective parental and resistant (Rociletinib-R) sublines were treated with 0.1, 0.2, 0.5, 1.0, 2.0, 5.0, and 10.0 µM dose of Rociletinib for 72 h. In addition, H1975 Rociletinib-R clones 1 and 2 sublines were treated with 0.002, 0.005, 0.01, 0.025, 0.05, 0.1, 0.25, and 0.5 μM Osimertinib for 72 h. Finally, parental and pooled, Osimertinib-resistant H1975 cells were treated with 0.05, 0.1, 0.2, 0.5, 1.0, 2.0, 5.0, and 10.0 μM Osimertinib. Percent cell viabilities were determined relative to respective DMSO-treated controls. The data in the GI50 columns represent means of three-four independent experiments.

To identify the effect of CFM compounds on the growth of the TKI resistant NSCLC cells we performed MTT assays as described in Figure 1A. As shown in Figure 1B, a dose of 5 μM and 10 μM of each of CFM-4 or CFM-4.16 inhibited growth of parental and Erlotinib-resistant HCC 827 cells. In the case of parental and Rociletinib-resistant H1975 cells, a significant loss of cell viability was also noted when treated with 1 μM, 5 μM, or 10 μM dose of respective CFMs (Figure 1C). Although both CFMs diminished viability of parental and TKI-resistant NSCLC cells, CFM-4.16 at the doses of 5 μM and 10 μM was generally more potent. These data further support our previous findings in TNBC and RCC cells wherein also, we observed CFM-4.16 exhibited increased potency in attenuating cell growth [21, 22]. Taken together, these studies underscore potential of the CFM class of compound(s) to inhibit drug-resistant cancers.

### CFM-4.16 suppresses activation of oncogenes in wild-type and TKI-resistant NSCLC cells

**Figure 2:**
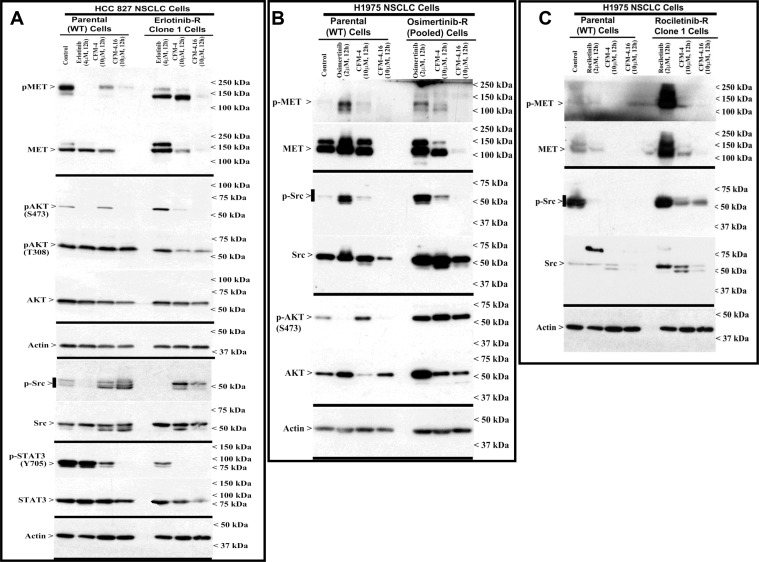
Altered oncogene expression in EGFR TKI-resistant NSCLC cells (**A**–**C**) Indicated parental or TKI-resistant NSCLC cells were either untreated (Control), treated with TKI, CFM-4, or CFM-4.16 for noted dose and time. Cell lysates were analyzed by Western blotting (WB) as in Methods for expression (total) or activation (phosphorylation) of oncogenes MET, AKT, Src, and STAT3. The western blot membranes were subsequently probed with anti-actin antibodies to assess equal loading. The presence of respective protein is indicated by an arrowhead on the left side of each blot. Approximate location of various molecular weight markers is indicated on the right side of each blot. kDa, kilodalton.

Development of resistance to TKIs erlotinib or gefitinib in NSCLC cells and patient tumors is often associated with abnormal expression and/or activation of oncogenic drivers MET, Alk, Vascular endothelial growth factor receptor (VEGFR), Fibroblast growth factor receptor (FGFR), Src and Abl TKs [23–25]. These TKs regulate development, progression, and metastasis of many cancers including NSCLCs, and often act as drivers of therapeutic resistance in NSCLCs and other cancers [23–25]. We performed immunoblot analyses to investigate molecular pathways involved in NSCLC growth suppression by CFMs, and to identify whether the CFMs targeted oncogenic tyrosine kinases and their signaling. We treated the wild-type and erlotinib-resistant HCC 827 NSCLC cells with erlotinib, CFM-4 or CFM-4.16, and the wild-type and rociletinib-resistant H1975 cells with rociletinib and the respective CFM compounds. In addition, we treated the osimertinib-resistant and wild-type H1975 cells separately with osimertinib, CFM-4 or CFM-4.16, and gemcitabine-resistant H23 NSCLC cells with gemcitabine or CFM compounds. Expression levels of Src and MET TKs were analyzed from the lysates through western blotting. As evident from Figure 2 and Supplementary Figure 1A, expression and/or activity of MET RTK was observed to be elevated in the TKI-resistant NSCLC cells. Activity and/or expression of Src was high in rociletinib or osimertinib-resistant H1975 cells but not in erlotinib-resistant HCC 827 cells. Treatment with CFM 4.16 reduced the activation and/expression of MET in parental, TKI resistant and gemcitabine resistant NSCLC cells (Figure 2A–2C, and Supplementary Figure 1A). Additionally, treatment with CFM-4.16 resulted in reduced expression and/or activation of Src in parental as well as TKI-resistant H1975 cells but not in parental or erlotinib-resistant HCC 827 cells (Figure 2). These data suggest that CFM-4.16 functions partly by modulating oncogenic kinase signaling pathways to inhibit cell growth. Interestingly, CFM-4.16 not CFM-4, attenuated the activation of STAT3, a transducer of signaling following activation of the EGFR and Src TKs [26, 27], in wild-type or erlotinib-resistant HCC 827 cells (Figure 2A). Furthermore, CFM-4.16 robustly inhibited oncogenic intracellular kinase Akt activity and/or expression in wild-type HCC 827, H1975 and H23 NSCLC cells (Figure 2A, 2B, and Supplementary Figure 1A). CFM-4.16 also inhibited Akt activation in erlotinib and gemcitabine-resistant, but not in osimertinib-resistant, NSCLC cells (Figure 2A, 2B, and Supplementary Figure 1A). These data collectively suggest that CFM-4.16 suppresses growth of NSCLC cells in part by reducing the activation/expression as well as downstream signaling of the key survival-regulating oncogenic drivers of drug resistance.

Among the mechanisms of development and maintenance of NSCLC resistance to EGFR TKIs Erlotinib or Gefitinib, amplification and/or activation of MET has been frequently reported in patients as well as in NSCLC cell models of TKI resistance [23, 24]. Accordingly, targeting of MET has been proposed as a potential strategy to overcome resistance to EGFR TKIs and “oncogenic addiction” of the NSCLCs [28]. The NSCLC cell models of resistance to third generation, allosteric inhibitors Rociletinib or Osimertinib that target mutant, constitutively active EGFR tyrosine kinase however have revealed involvement of EGFR-dependent as well as independent mechanisms [5, 29, 30]. Consistent with these observations, our immunoblot studies revealed overexpression and activation of MET and Src kinases in erlotinib-resistant HCC 827 cells (see Figure 2A, and Supplementary Figure 2A), while a moderate upregulation of EGFR levels was noted in rociletinib-resistant H1975 cells (Supplementary Figure 2B). We next tested whether CFM-4.16 could have a superior effect in suppressing the growth of the drug resistant cells when used in combination with other well known MET and Src inhibitors. For this study we used FDA-approved Dasatinib, a multi-targeted orally administered inhibitor of RTKs and Src [31] and Tivatinib, an investigational orally administered, highly selective inhibitor of the MET RTK [32]. As shown in Supplementary Figure 2B, treatment of parental or erlotinib-resistant HCC 827 cells with Dasatinib or Tivatinib, following pre-treatment with CFM-4.16, resulted in significantly reduced viabilities when compared with the cells that were treated with each compound separately. Interestingly, as also shown in Supplementary Figure 2D, pre-treatments of wild-type or rociletinib-resistant H1975 cells with CFM-4.16 followed by addition of Gefitinib (EGFR TKI) or Rociletinib also resulted in significantly reduced viabilities when compared with the cells treated with each compound separately. These proof-of-principle findings suggest that CFM-4.16 can sensitize TKI-resistant NSCLC cells to inhibition by TKIs that target EGFR or other oncogenic driver tyrosine kinases.

### CFM-4.16 promotes apoptosis in parental and drug-resistant NSCLC cells through activation of c-Jun N-terminal kinase (JNK), stress-activated protein kinases p38, and enhancing expression of CCAR-1/CARP-1

**Figure 3:**
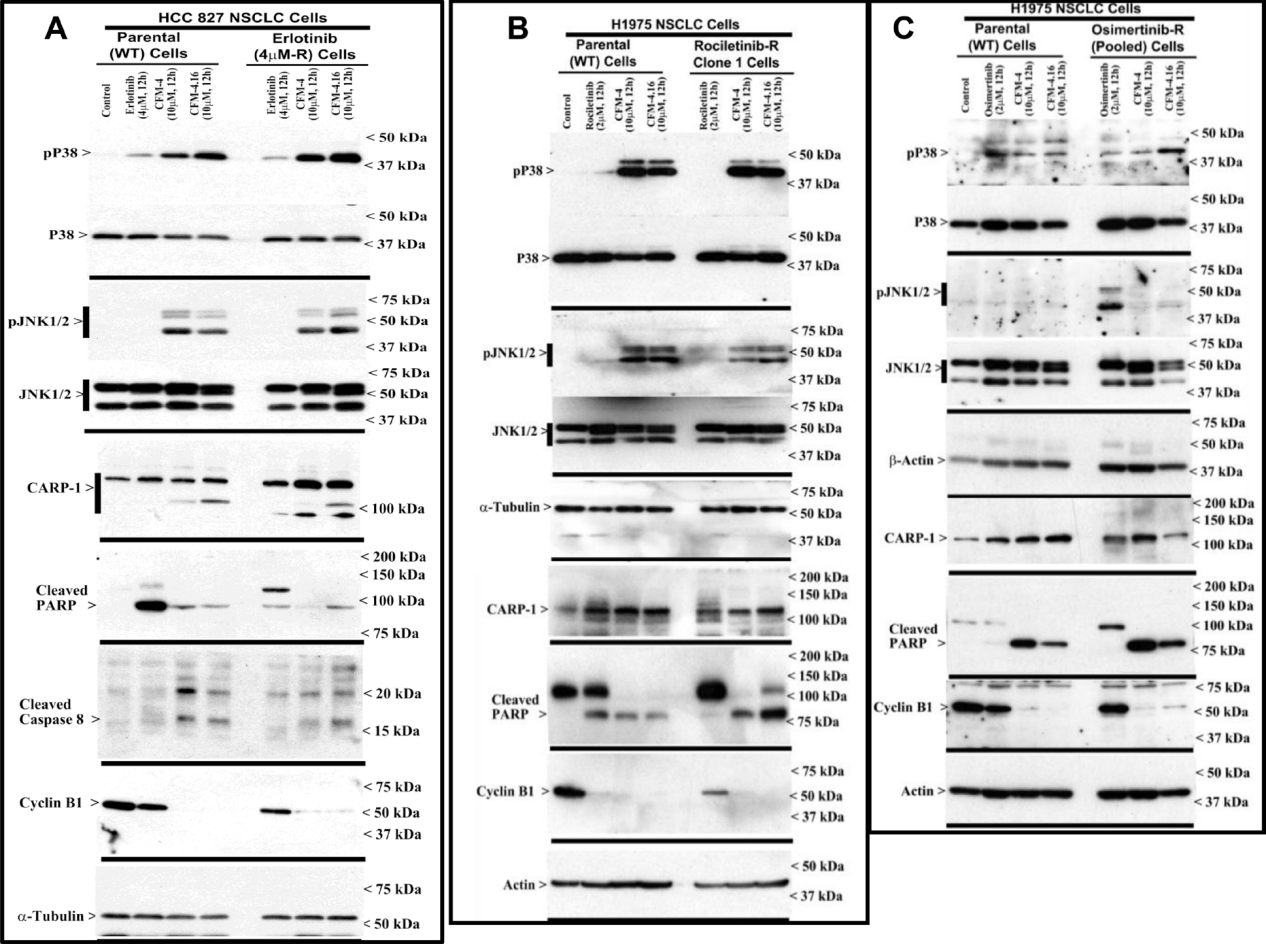
CFM-4.16 stimulates apoptosis in parental and TKI-resistant NSCLC cells in part by upregulating pro-apoptotic CARP-1 and activating SAPKs (**A**–**C**) Indicated parental and TKI-resistant NSCLC cells were either untreated (Control), treated with TKI, CFM-4, or CFM-4.16 for noted dose and time. Cell lysates were analyzed by Western blotting (WB) as in Methods for levels of CARP-1, cyclin B1, cleaved PARP and caspase-8, and activation (phosphorylation) of pro-apoptotic p38 and JNK1/2 SAPKs. The western blot membranes were subsequently probed with anti-actin or α-tubulin antibodies to assess equal loading. The presence of respective protein is indicated by an arrowhead on the left side of each blot. Approximate location of various molecular weight markers is indicated on the right side of each blot. kDa, kilodalton.

We have previously reported that the apoptosis signaling by the chemotherapeutic drugs, Doxorubicin, Gefitinib and Etoposide is mediated through CARP-1 [7, 8]. Expression of CARP1 was required for mediating the apoptotic/inhibitory signaling induced by these drugs and also our experimental CFM analogs in TNBC and RCC cells [21, 22]. Since CFM-4.16 robustly inhibited the growth of wild-type and TKI-resistant NSCLC cells (Figure 1), we further investigated whether expression of CARP1 was necessary for CFM-induced growth inhibition and the molecular mechanisms involved. We observed that equimolar (10 μM) dose of CFM-4 or CFM-4.16 induced CARP-1 expression and activation of pro-apoptotic, stress-activated protein kinases (SAPKs) in the wild-type and drug (TKI or gemcitabine)-resistant NSCLC cells (Figure 3, and Supplementary Figure 1B). In addition to activation of SAPK, treatments with CFMs also stimulated cleavage of PARP, activation of caspase 8, and reduction in levels of mitotic cyclin B1 in the parental and resistant NSCLC cells (Figure 3, and Supplementary Figure 1B). These data suggest that CFMs suppress NSCLC cell growth in part by inducing apoptosis.

To further elucidate TKI resistance molecular pathways downstream of the RTKs, we conducted additional immunoblot analyses utilizing the parental and TKI-resistant NSCLC cells listed in Table 1. These analyses revealed activation of mitogen-activated protein kinase p38α/β, and NF-κB subunit p65/RelA in the NSCLC cells that are resistant to Erlotinib or Rociletinib (Supplementary Figure 3A). Activation of NF-κB subunit p65/RelA would be consistent with well-documented roles of proliferation and survival-promoting NF-κB signaling that is often activated down-stream of a number of activated driver RTKs. The intriguing activation of p38α/β SAPK however in the untreated drug-resistant NSCLC cells as well as in the parental or TKI-resistant NSCLC cells that were treated with CFMs (see Figure 3 and Supplementary Figures 1 and 3) would suggest for known biphasic growth and stress signaling roles, respectively, of p38α/β kinase. Environmental stresses and inflammatory mediators activate the core MAPKs that consist of ERK1/2, JNK1/2/3, and p38α/β/γ/δ kinases [33]. MAPKs orchestrate the recruitment of gene transcription, protein biosynthesis, cell cycle control, apoptosis, and differentiation processes. The p38 and JNKs also regulate stress-dependent inhibition of cellular growth and thus are also known as stress-activated protein kinases (SAPK). A number of pharmacologic inhibitors p38α/β and JNKs have been discovered and characterized, and tested for use in pre-clinical and clinical settings with respect to cancer [34] and other autoimmune disorders, particularly rheumatoid arthritis [35]. Expectedly, treatments with pharmacological inhibitors of p38α/β (Doramapimod/BIRB796 or Losamapimod), resulted in attenuation/blockage of p38 activation in TKI-resistant NSCLC cells in a dose-dependent manner, while pre-treatment with Doramapimod or Losamapimod re-sensitized TKI-resistant NSCLC cells to respective TKIs (Supplementary Figure 3B, 3C). These proof-of-concept studies underscore a significant and novel mechanism of TKI resistance, and reveal presence of signaling “node” with potential to permit development of additional targeting strategies for treatment and management of RTK-driven resistant NSCLCs.

**Figure 4:**
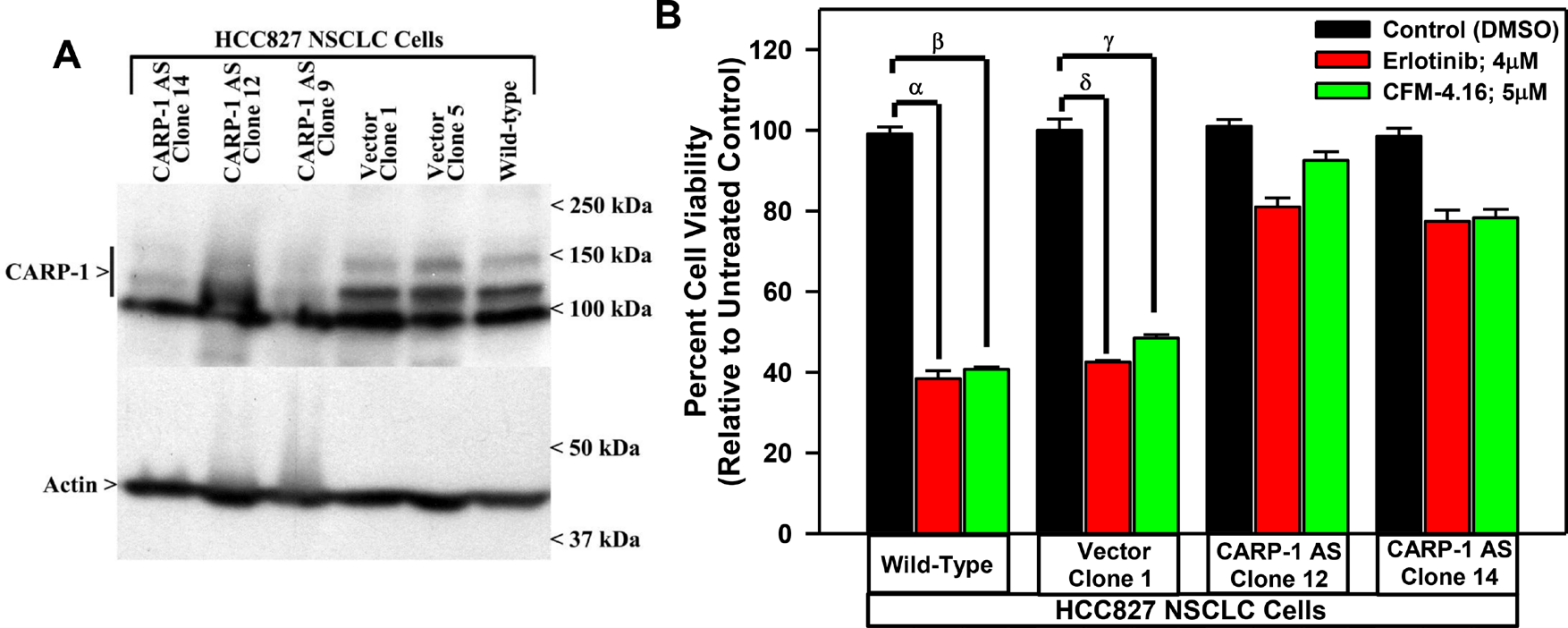
Knockdown of CARP-1 blocks CFM-4.16 effects (**A**) Cells were either untransfected, transfected with the pcDNA3/Hygro vector plasmid or plasmid expressing CARP-1 antisense, and stable, hygromycin-resistant cells were generated and characterized as detailed in methods. Cell lysates from indicated stable cell lines were subjected to WB analysis as in Figure 3 for levels of CARP-1 and actin. (**B**) The Indicated vector or CARP-1 antisense expressing NSCLC sublines were treated with DMSO (Control), noted doses of CFM-4.16, or Erlotinib for 24 h. Cell viability was determined by MTT assay. The histogram columns represent means of three independent experiments; bars, S.E. α, β, γ, δ, *p* ≤ 0.05 for the Erlotinib or CFM-4.16-treated wild-type or the vector expressing subline relative to their respective DMSO-treated control.

To investigate whether CARP-1 expression is necessary for the CFM mediated cell growth inhibition of the NSCLC cells, we generated stable sublines of HCC827 expressing plasmid encoding CARP-1 antisense or its vector as described previously [7]. The stable cell lines generated were then characterized, by measuring the levels of CARP-1 as shown in Figure 4A. In the cells expressing the anti-sense plasmid, CARP-1 expression was reduced compared to the cells expressing the vector and the parental cells. The characterized clones were then treated with CFM-4.16 and TKI Erlotinib and the viabilities of the cells were measured. We observed that in the HCC827 cells expressing CARP-1 antisense, the inhibitory effects of CFM-4.16 and Erlotinib was significantly reduced compared to the vector plasmid expressing cells (Figure 4B). These findings suggest that the CFMs inhibit growth of parental as well as drug resistant NSCLC cells, and the expression of CARP-1 is necessary to mediate this effect.

### CFM-4.16 functions synergistically with B-RAF targeting to inhibit growth of various cancer cells

In light of our prior studies demonstrating superior inhibition of *in vitro* and *in vivo* growth of TNBC cells by a combination of Adriamycin and CFM-4.16 [21], we next investigated whether CFM-4.16 would enhance effectiveness of other, targeted or chemo-therapeutics that are currently used in clinic. To test this hypothesis, we chose compounds that target cell cycle (CDK4/6 inhibitor Palbociclib), DNA repair pathway (PARP inhibitor Olaparib), cell metabolic signaling (mToR1 inhibitor Everolimus), and oncogenic cell growth and survival signaling (B-Raf inhibitors Sorafenib and Dabrafenib). The U.S. Food and Drug Administration (USFDA) recently granted regular approval to Palbociclib (IBRANCE^®^, Pfizer Inc.) for the treatment of hormone receptor (HR) positive, human epidermal growth factor receptor 2 (HER2) negative advanced or metastatic breast cancer in combination with an aromatase inhibitor as initial endocrine based therapy in postmenopausal women [36]. USFDA previously approved Olaparib as monotherapy in ovarian cancer patients with germline BRCA1 mutation [37], and recently expanded Olaparib as maintenance treatment for patients with recurrent epithelial ovarian, fallopian tube, or primary peritoneal cancer who are having partial or complete responses to platinum-based chemotherapy. While Sorafenib serves as one of the frontline therapy for treatment of thyroid, liver, and kidney cancers, Everolimus is a frontline therapy to treat select pancreatic, breast and brain as well as renal cancers including a subset of renal cancers where prior treatments with Sorafenib or Sunitinib had failed. Last, but not least, although Dabrafenib was earlier approved by USFDA for treatment of B-Raf V600E mutation-positive un-resectable or metastatic melanoma, recently it was also approved for treatment of B-Raf V600E mutation-positive NSCLC in combination with MEK-targeting therapeutic Tramitinib [38].

**Figure 5:**
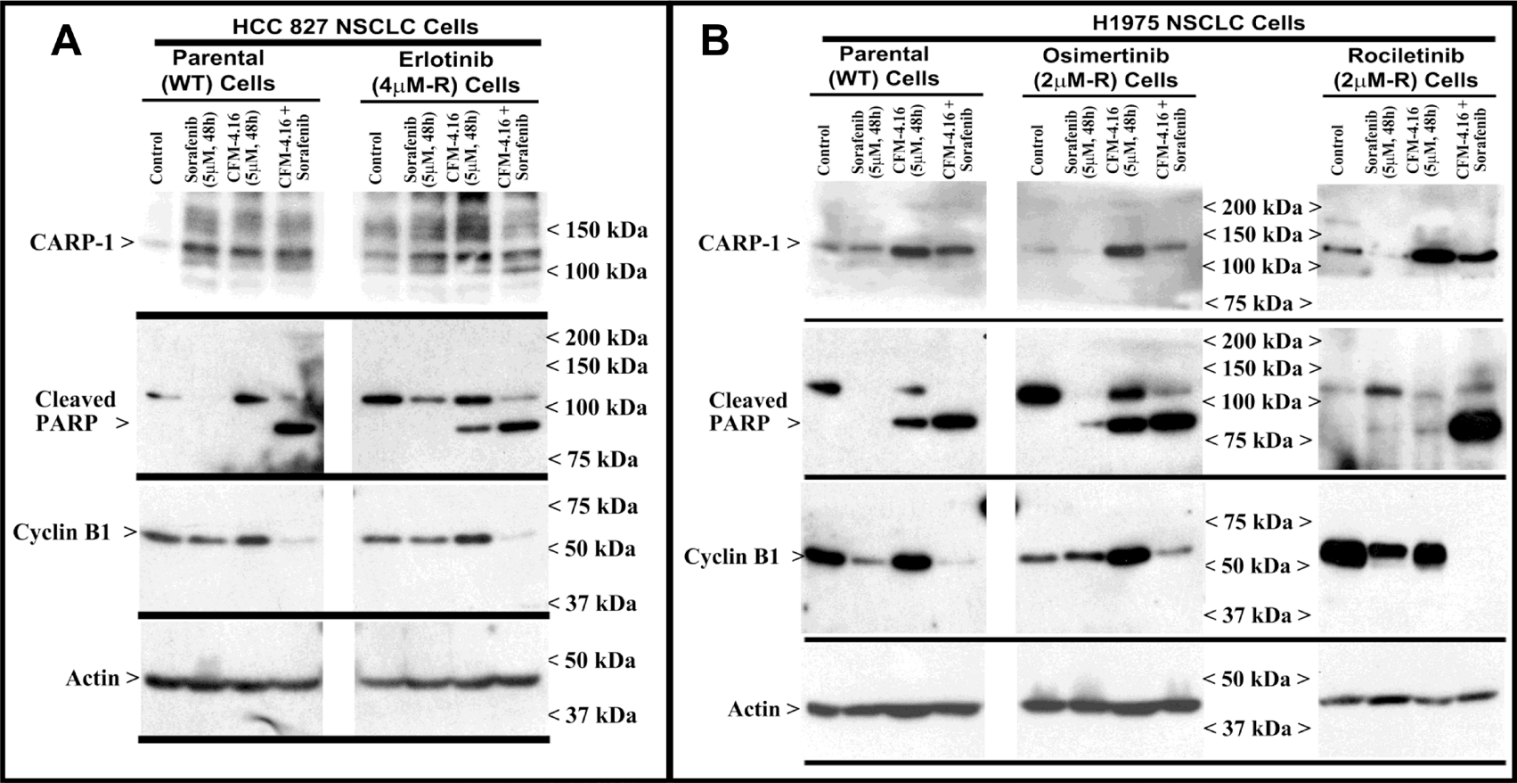
Apoptosis induction in parental and TKI-resistant NSCLC cells following treatments with CFM-4.16 and Sorafenib (**A**, **B**) Indicated parental and TKI-resistant NSCLC cells were either untreated (Control), treated with Sorafenib, CFM-4.16, or a combination of Sorafenib and CFM-4.16 for noted dose and time. Cell lysates were analyzed by Western blotting (WB) as in Methods for levels of CARP-1, cleaved PARP, cleaved RIPK1, activated and total Akt and B-Raf kinases, and expression of cyclin B1 as in Figure 3. The western blot membranes were subsequently probed with anti-actin antibodies to assess equal loading. The presence of respective protein is indicated by an arrowhead on the left side of each blot. Approximate location of various molecular weight markers is indicated on the right side of each blot. kDa, kilodalton

**Figure 6:**
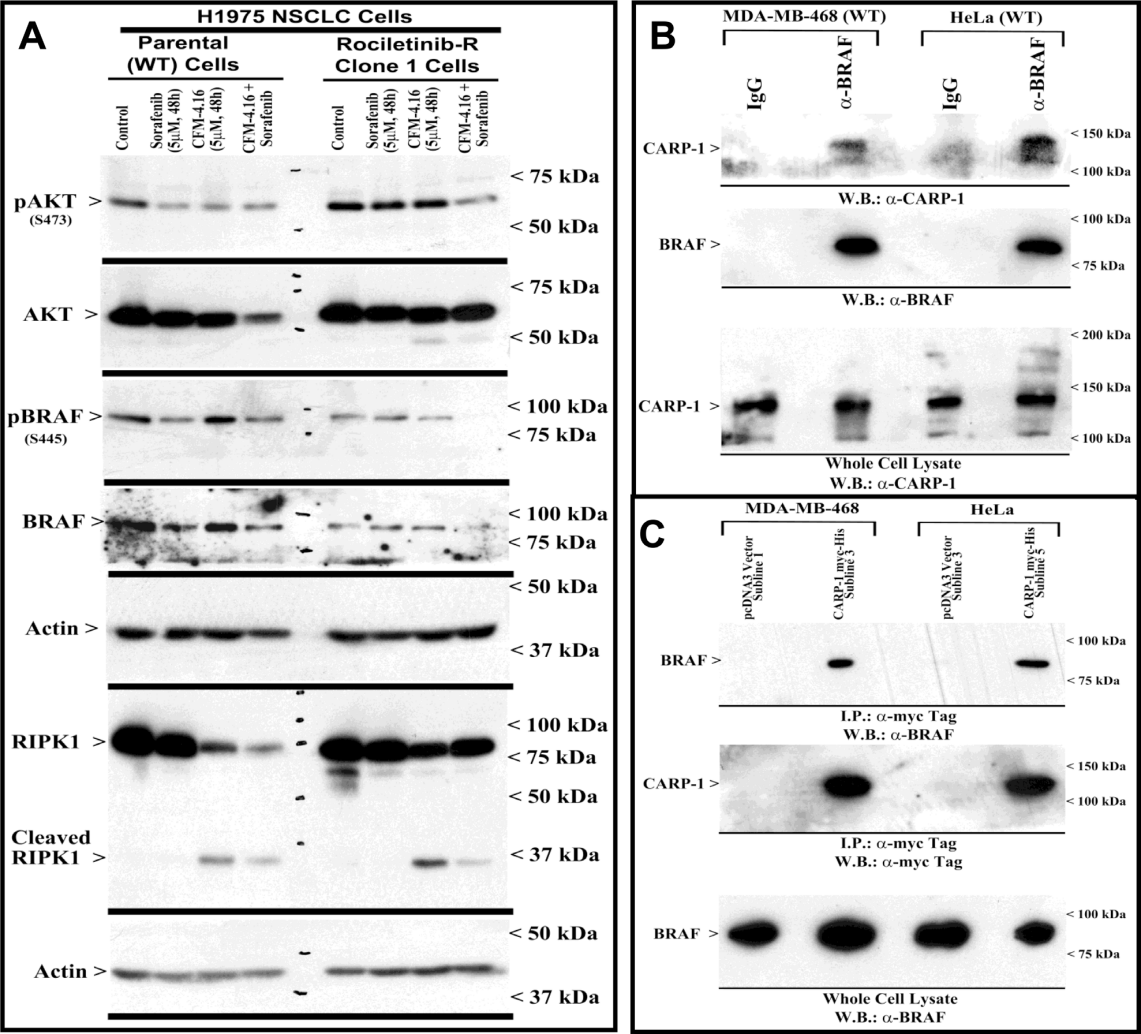
CFM-4.16 and Sorafenib combination inhibits B-Raf activity, and CARP-1 interacts with B-Raf (**A**) Indicated parental and TKI-resistant NSCLC cells were either untreated (Control), treated with Sorafenib, CFM-4.16, or a combination of Sorafenib and CFM-4.16 for noted dose and time. Levels of actin, total and cleaved RIPK1, activated and total Akt and B-Raf kinases were analyzed by western immunoblotting essentially as in Figure 5. (**B**, **D**) Approximately 1mg of cell lysate from each of the indicated cells was subjected to immunoprecipitation using anti-B-Raf (B) or anti-myc-tag (**C**) antibodies. The immunoprecipitates were then analyzed by WB. In B, the membrane was probed with anti-CARP-1 (α2) antibodies (7) or B-Raf antibodies while the membrane in C was probed with B-Raf or myc-tag antibodies. The presence of respective protein is indicated by an arrowhead on the left side of each blot. Approximate location of various molecular weight markers is indicated on the right side of each blot. kDa, kilodalton.

Our MTT-based cell viability analyses revealed that each of the targeted therapeutic or CFM-4.16 inhibited viabilities of TNBC, NSCLC, and RCC cells *in vitro* following treatments with respective compounds. Further analyses of data revealed that cells treated with CFM-4.16 in combination with Olaparib or Palbociclib were additive in their efficacy with combination index (CI) values ≥ 0.8 (Table 2). The combinatorial treatment of Everolimus and CFM 4.16 however, elicited an additive CI for the NSCLC and RCC cells, their CI values however were <0.8 for the TNBC cells suggesting a possible synergy in their mechanisms of action (Table 2). Interestingly, CFM-4.16 in combination with Sorafenib or Dabrafenib consistently elicited CI values that were <0.8 for the TNBC, NSCLC and RCC cells with the exception of H1299 NSCLC and UOK262 RCC cells where CI values for CFM-4.16 and Sorafenib but not Dabrafenib combination were ∼0.8 (Table 2). Moreover, with reference to the NSCLC model, although treatments with Sorafenib or CFM-4.16 increased expression of CARP-1in parental and Erlotinib-resistant HCC827 cells, CFM-4.16 but not Sorafenib stimulated CARP-1 expression in the parental and Rociletinib- or Osimertinib-resistant H1975 cells (Figure 5A, 5B). However, the combinatorial treatment of Sorafenib and CFM-4.16 failed to induce further CARP-1 increase in these cells. Of note is that although Sorafenib or CFM-4.16 also stimulated a moderate PARP cleavage and loss of cyclin B1 in the parental and their respective, TKI-resistant counterparts, the combined presence of Sorafenib and CFM-4.16 elicited a rather robust cleavage of PARP and RIPK1 proteins as well as loss of cyclin B1 in the parental and TKI-resistant cells (Figures 5A, 5B, 6A). A combination of both the compounds also induced a robust decline in activities of oncogenes Akt and B-Raf in parental as well as TKI-resistant cells (Figure 6A). Interestingly, our co-immunoprecipitation experiments revealed an interaction between the B-Raf and CARP-1 proteins (Figure 6B, 6C; Supplementary Figure 4). Thus, although CARP-1 is part of the B-Raf proteome, it is likely that CFM-4.16 binding with CARP-1 leads to CARP-1 increase and resultant stress while Sorafenib targeting of B-Raf further inhibits oncogenic/survival signaling and together a combination of both the compounds likely cause elevated levels of stress with consequent robust cell growth inhibitory effects. We have previously found CARP-1 is a co-activator of the anaphase-promoting complex cyclosome (APC/C) E3 ubiquitin ligase (9). Whether and to the extent CARP-1 interaction with B-Raf is involved in ubiquitin-dependent altered expression of B-Raf remain to be clarified. Altogether, our findings in Table 2 and Figures 5 and 6 suggest that CFM-4.16 could be a novel, sensitizer for Raf targeting therapeutics in various types of cancers.

**Table 2:** Combination index (CI) values of CFM-4.16 plus various targeted therapeutics

	Combination Index Drug + CFM-4.16					
Cell Lines	Sorafenib	Everolimus	Olaparib	Palbociclib	Ulixertinib	Dabrafenib
MDA-MB-231 (TNBC)	0.256	0.317	1.077	0.557	0.393	0.4231
HCC-1937 (TNBC)	0.84910	0.44371	ND	ND	0.715	0.7012
Hs-578T (TNBC)	0.6512	0.5121	ND	ND	0.733	0.4653
MDA-MB-468 (TNBC)	0.689	0.699	1.305	0.811	0.655	0.498
HCC-70 (TNBC)	0.68	0.698	1.305	0.811	ND	0.626
HCC-1806 (TNBC)	0.69614	0.27961	ND	ND	0.554	0.4121
H460 (NSCLC)	0.147	0.313	1.194	0.818	ND	0.512
H1299 (NSCLC)	0.84	0.79623	1.59868	1.07999	ND	0.612
HCC-827 (NSCLC)	0.35800	0.81829	1.1987	0.9213	ND	0.15672
H-1975 (NSCLC)	0.47810	0.54892	1.1	0.9542	ND	0.21412
A498-wild type (RCC)	0.53	0.7	1.0978	0.7	ND	0.13850
A498-Everolimus Resistant type (RCC)	0.6	1.3	1.121	0.971	ND	0.677
UOK262-wild type (RCC)	0.76	0.67	0.9878	0.897	ND	0.234
UOK262-Everolimus Resistant type (RCC)	0.8	1.45	1.312	1.0023	ND	0.587
MDA-MB-231 (TNBC)	0.53	0.313	1.194	0.818	ND	0.512

Abbreviations: Sorafenib, Dabrafenib: B-Raf Inhibitors. Everolimus: mToR Inhibitor. Olaparib: PARP Inhibitor. Palbocicilib: CDK4/6 Inhibitor. Ulixertinib: ERK1/2 Inhibitor. ND: Not Done; TNBC: Triple-negative Breast Cancer; NSCLC: Non-small Cell Lung Cancer; RCC: Renal Cell Carcinoma.

### CFM-4.16 inhibits migration and three-dimensional growth of the parental and TKI-resistant NSCLC cells

**Figure 7:**
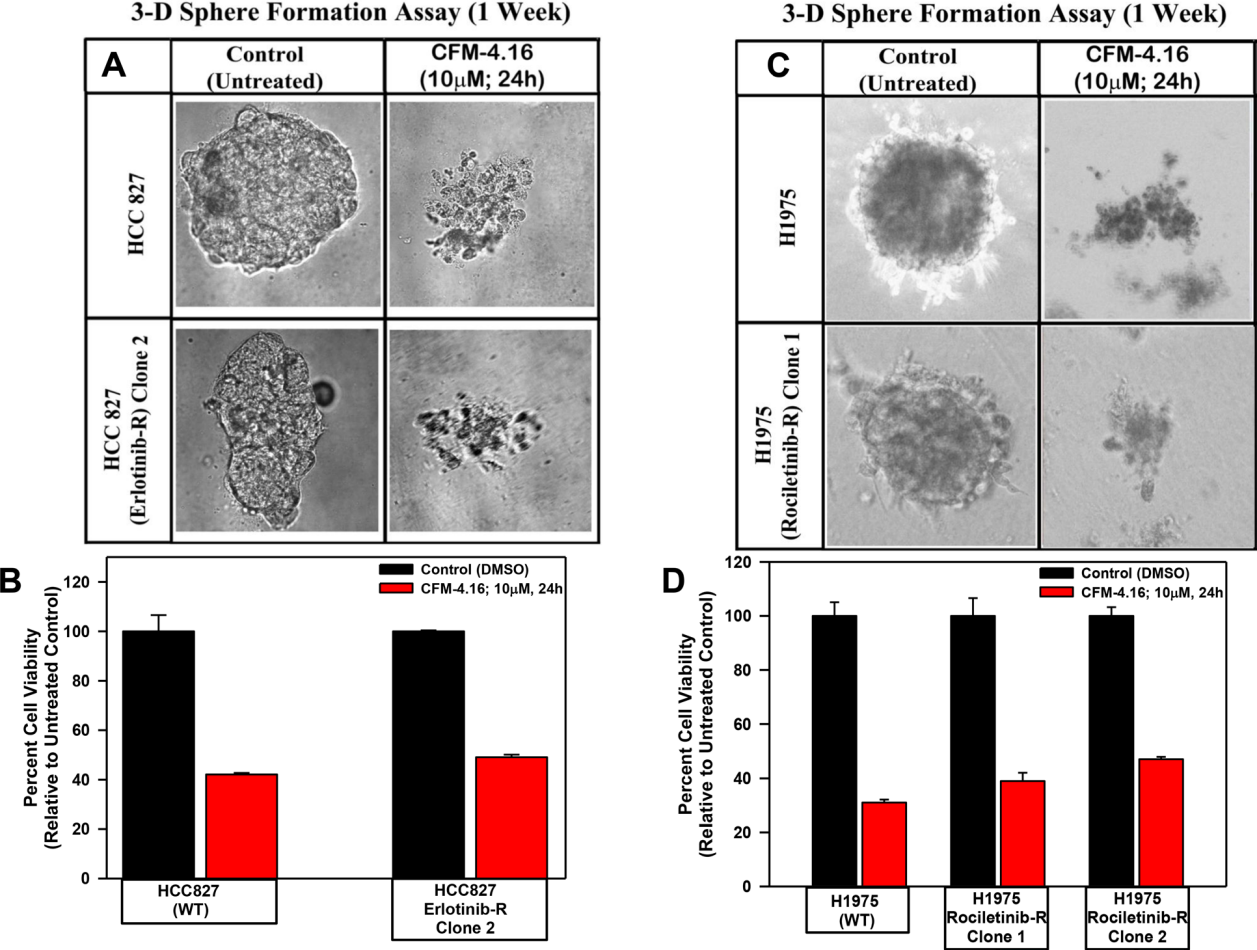
CFM-4.16 inhibits growth of NSCLC spheres derived from parental and TKI-resistant cells Parental and TKI-resistant NSCLC cells were grown as spheres as detailed in Methods. The sphere cultures were either untreated (Control) or treated with CFM-4.16 for noted dose and time. The untreated and treated spheres were then photographed (**A**, **C**) or the cells were subjected to MTT-based viability assay as in Figure 1 (**B**, **D**). Representative photomicrographs of untreated and CFM-4.16 treated spheres are shown in panels A and C. The histograms in panels B and C represent means of three independent experiments, bars, S.E.

Since CFM-4.16 robustly disrupted integrity of the tubules formed by human umbilical vein endothelial cells, suppressed TNBC cell growth and migration as colonies in soft agar and 3-dimensional cultures *in vitro*, we tested whether CFM-4 or -4.16 compounds will also influence the biological properties of migration and 3-dimensional growth of Erlotinib- or Gemcitabine-resistant NSCLC cells. We observed that treatment with CFM-4 or CFM-4.16 prevented the growth of parental and Erlotinib or Gemcitabine-resistant NSCLC cells into the areas of wound caused by a scratch (Supplementary Figure 5A–5E). We also noted significant attrition in size and number of colonies formed by the parental and Erlotinib or Gemcitabine-resistant NSCLC cells in soft agar upon treatment with CFM-4 or CFM-4.16 (Supplementary Figure 6A–6C). We have previously observed significant reduction in the growth of mammospheres and shperoids derived from TNBC and RCC cells, respectively, upon treatments with CFM-4.16 [21, 22] We investigated whether a similar inhibitory effect occurs on spheroids derived from NSCLC cells when treated with CFM-4.16. As depicted in Figure 7, the parental HCC 827, H1975 cells and also their respective, TKI-resistant sublines formed NSCLC spheroids. Similar to our previous observations in TNBC and RCC models, treatment with CFM-4.16 resulted in disintegration of spheres of both the parent as well as TKI resistant human NSCLC cells (Figure 7).

### Combinatorial treatment of CFM-4.16 NLF with Sorafenib causes superior inhibition of xenografted, TKI rociletinib-resistant NSCLC tumors

**Figure 8:**
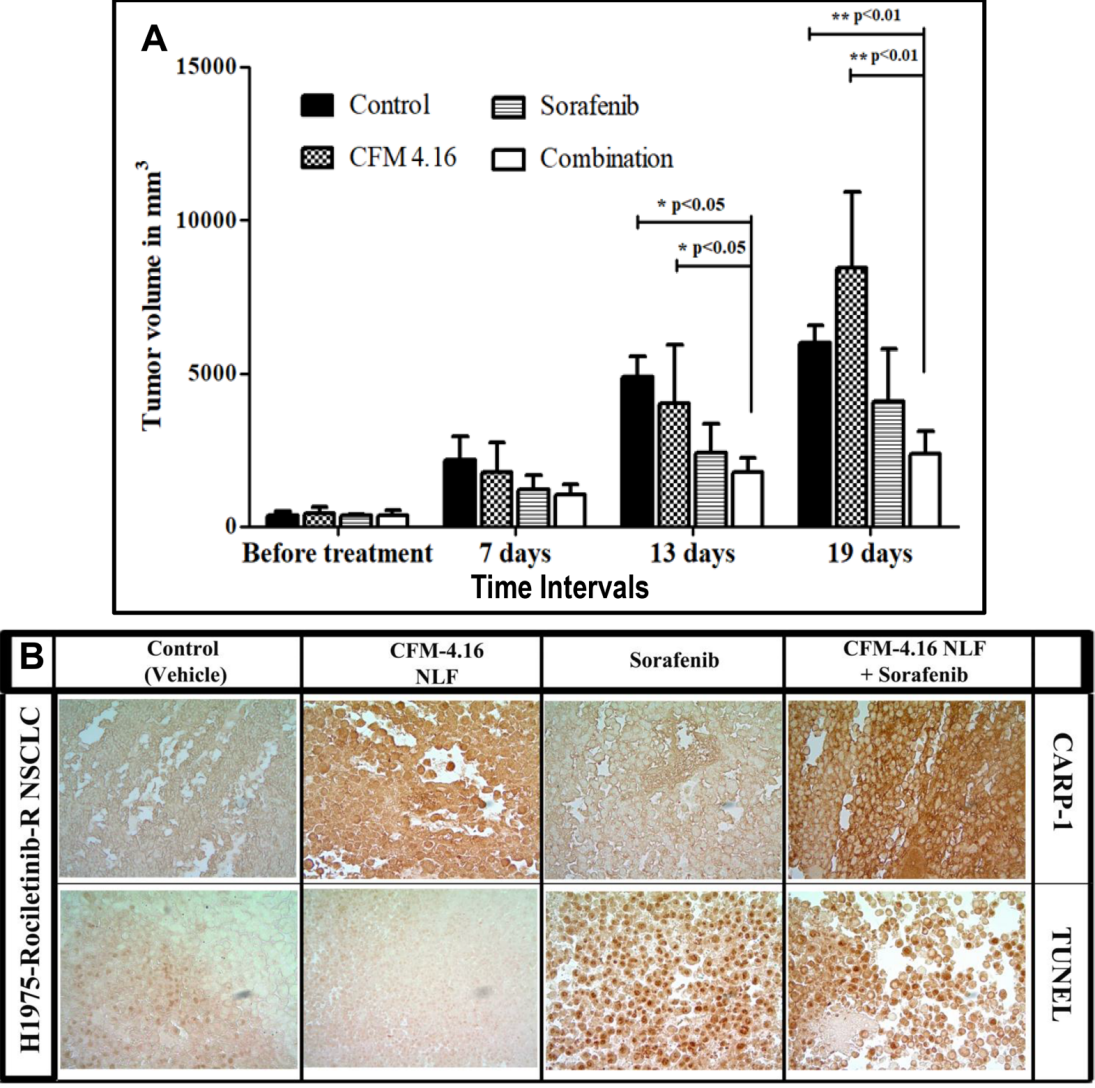
Nano-lipid formulation of CFM-4.16 in combination with Sorafenib inhibits growth of TKI-resistant NSCLC cell-derived xenografts (**A**) Histogram showing tumor volume of the vehicle-treated (indicated as Control), CFM-4.16-NLF, Sorafenib, or CFM-4.16 plus Sorafenib-treated, Rociletinib-resistant NSCLC (H1975) xenograft-bearing animals. The xenograft establishment, treatment and analysis procedures were carried out essentially as detailed in Methods. The columns represent average values from a total of six animals in respective group, bars, SE, significant where ^**^p = 0.01 vs Control. (**B**) CFM-4.16-NLF and Sorafenib treatments (po) induce CARP-1 expression and apoptosis in NSCLC tumor xenografts. NSCLC tumor xenografts generation and animal treatments were as in Methods. Representative tumor tissues from two animals each from the Control or treated groups were fixed in formalin, paraffin embedded, processed, and subjected to immuno-staining as detailed in Methods. Photomicrographs (400 × magnification) are shown for apoptosis (by TUNEL assay), and levels of CARP-1 protein as noted in methods.

We have previously developed and tested nano-lipid formations (NLFs) of CFM-4 and CFM-4.16 to address the issues of poor bioavailabity and poor aqueous solubility [20–22]. These NLFs resulted in significant improvements in overall bioavailabilities of CFM-4 and CFM-4.16 [20, 21]. On the basis of our current findings in Table 2 that suggested a synergistic NSCLC inhibitory mechanism of CFM-4.16 when used in combination with B-Raf targeting, we investigated the anti-tumor efficacy of CFM-4.16 NLF in combination with Sorafenib, *in-vivo*. Nude mice bearing Rociletinib-resistant NSCLC H1975 orthotropic xenograft tumors were treated with CFM-4.16 NLF, Sorafenib, or a combination of both by oral gavage as described in methods and our published protocols [20, 21]. As depicted in Figure 8A, the control (placebo) and CFM-4.16 NLF groups did not show significant tumor growth inhibition. Although oral administration of Sorafenib showed reduction in tumor volume over a 13 and 19-day period, the combinatorial treatment of CFM-4.16 NLF and Sorafenib resulted in significantly reduced tumor volume when compared with treatments of compounds on their own. Our result showed that from all the formulations evaluated, combination of CFM-4.16 NLF and Sorafenib was able to significantly reduce tumor burden after 13 (*p <* 0.05) and 19 (*p <* 0.01 and *p <* 0.001) days when compared with the control group and group of animals treated with CFM-4.16 NLF alone. The other formulations could not decrease the tumor burden significantly when compared with the control. We then performed immuno-histological analysis of a representative NSCLC tumor from the animals treated with placebo (control), CFM-4.16 NLF, Sorafenib, or combination of CFM-4.16 NLF and Sorafenib. We observed increased staining for TUNEL and CARP-1 protein in the tumors from the treated animal when compared with the tumor from the control, placebo-treated animal (Figure 8B). Of note is fact that consistent with our data in Figure 5B where a higher levels of CARP-1 were noted in the Rociletinib-resistant H1975 cells, staining for CARP-1 were rather intense in the tumor derived from the animal treated with CFM-4.16 NLF. The tumor from the animal treated with a combination of CFM-4.16 NLF and Sorafenib had robust CARP-1 levels when compared with the tumor derived from the animal treated with Sorafenib only. The data in Figure 8 collectively demonstrate that CFM-4.16 enhances efficacy of Sorafenib to inhibit TKI-resistant NSCLC tumor *in vivo*. These findings would be consistent with our current *in vitro* observations as well as our previous studies where CFM-4.16 stimulated apoptosis in a variety of cancer cell types including those of TNBC and RCC origins [21, 22].

## DISCUSSION

We had previously identified CARP-1/CCAR1, a perinuclear phosphoprotein, which is a biphasic regulator of chemotherapy-induced apoptosis as well as steroid-induced activation of steroid/thyroid nuclear receptors [7, 8, 11, 12]. Our recent chemical biology studies culminated in identification of small molecule compounds termed CARP-1 functional mimetics (CFMs) that bind with CARP-1 and stimulate apoptosis in various cancer cells [9, 17–22]. CFM-4 analog -4.16 elicited superior inhibition of only the TNBC cells *in vitro* and TNBC cell-derived xenografted tumors *in vivo* when administered in combination with the chemotherapeutic Adriamycin [21]. CFM-4.16 was also superior in inhibiting growth of parental and mammalian target of rapamycin (mToR) inhibitor Everolimus-resistant renal cancer cells (RCC) *in vitro* and *in vivo* [22]. Although, CFM compounds including CFM-4.16 are not water-soluble and consequently have poor bioavailability, nano-lipid formaulations significantly enhanced bioavailabilities of these compounds following their oral or intravenous administration when compared with similarly administered free compounds in rodents [20–21]. Given that CARP-1 is also a transducer of inhibitory signaling following targeting of EGFR [8], and the fact that previous studies also demonstrated CARP-1 antagonism of EGFR signaling in *C. elegans* [16], prompted us to investigate whether we could exploit CARP-1 signaling for inhibition of cancers that develop resistance to EGFR-targeted therapeutics. We tested this hypothesis in the current study in conjunction with a subset of NSCLC cells. These established NSCLC cell models are representative of patient tumors that often harbor mutant, constitutively active EGFR where treatments with EGFR TKIs are frontline options, and development of resistance to various EGFR-targeting TKIs continues to be a challenge in clinical management of this disease.

A wealth of recent investigations have reported a variety of redundant, EGFR-independent mechanisms of NSCLC resistance to EGFR TKIs in clinic as well as in pre-clinical NSCLC models. Consistent with these crucial mechanistic findings of development of EGFR TKI-resistant NSCLCs, additional therapies targeting resistant NSCLCs have proven beneficial in clinic. Yet the progression-free survival in patients with resistant NSCLC remains unacceptably low. To begin to test potential of CFM compounds in inhibiting TKI-resistant NSCLCs, we first obtained and characterized EGFR mutant NSCLC cells with induced resistance to chronic presence of TKIs. We chose HCC 827 cells with EGRRΔ19 mutation and exposed these cells to TKI Erlotinib. Additionally, we exposed the H1975 cells with EGFR L858R and the ‘gatekeeper’ T790M mutations to TKIs Rociletinib or Osimertinib. As shown in Table 1, we obtained a number of TKI-resistant NSCLC cells that were further characterized. Consistent with a number of prior reports [4, 5, 28], Erlotinib resistant HCC827 cells had elevated levels and activities of MET and its downstream Src and Akt kinases. Rociletinib-resistant H1975 cells also had elevated expression and activities of MET. Moreover, in agreement with prior findings [29], we found a moderate increase in expression but not activation of EGFR in these Rociletinib-resistant NSCLC cells. Osimertinib-resistant H1975 cells, however, had increased expression and activities of Src and Akt kinases. Together with significantly elevated IC50 values that were noted for the respective TKI-resistant sublines that are summarized in Table 1, our studies suggest for emergence of robust, TKI-resistant phenotypes of EGFR-mutant NSCLC cells. Importantly, our data in Supplementary Figure 2, also suggest a proof-of-concept targeting of the parental or the Erlotinib-resistant NSCLC cells by CFM-4.16 in combination with Src targeting (Dasatinib) or MET targeting (Tivatinib). Similarly, although CFM-4.16 in combination with Rociletinib elicited higher efficacy in inhibiting parental or rociletinib-resistant H1975 cells, CFM-4.16 in combination with EGFR TKI Gefitinib caused greater inhibition of Rociletinib-resistant H1975 cells when compared with their parental counterparts. These preliminary studies suggest that CFM-4.16 compound could potentially enhance effectiveness of current TKIs in inhibiting parental and, more importantly, EGFR TKI-resistant, NSCLC cells.

A novel aspect of our current studies is the finding that the compound CFM-4.16 functions synergistically with therapeutics that target Raf kinases (Sorafenib or Dabrafenib; Table 2). Interestingly, we noted synergism between CFM-4.16 and Sorafenib or Dabrafenib for a number of NSCLC, TNBC as well as RCC cells. Sorafenib is a multikinase inhibitor that targets RTKs such as VEGFR and PDGFR, as well as the C- and B-Raf kinases [39, 40]. The fact that Dabrafenib, a B-Raf kinase inhibitor that is used as single agent treatment for patients with B-Raf V600E mutation-positive advanced melanoma as well as in combination with MEK inhibitor Trametinib for advanced or metastatic NSCLCs [38, 41, 42], also synergized with CFM-4.16 would strongly argue for Raf targeting being synergistic with CFM-4.16 in a variety of cancer cells. Since CFM-4 functions in part by binding with CARP-1 and stimulating CARP-1-mediated stress and apoptotic signaling [9, 10, 20–22], the synergistic actions of CFM-4.16 and Sorafenib then are likely to activate CARP-1-dependent stress and apoptosis together with Raf targeting that will impede proliferation and survival. Our findings in Figure 5 further highlight this synergistic aspect of CFM-4.16 and Sorafenib functions. Here CFM-4.16 or Sorafenib treatments cause a moderate increase in PARP cleavage as well as decline in levels of mitotic cyclin B1 in both the parental and EGFR TKI-resistant NSCLC cells. However, we noted a robust cleavage of PARP and RIPK1 proteins, diminished activities of oncogenic Akt and B-Raf kinases, as well as cyclin B1 loss in CFM-4.16 plus Sorafenib-treated cells.

Last, but not least, we tested the potential of CFM-4.16 in combination with Sorafenib to inhibit growth of xenografted, Rociletinib-resistant H1975 NSCLC cell-derived orthotopic tumors in immunocompromised (nude) mice. Here, we administered Sorafenib, CFM-4.16 NLF, or a combination of both the agents by oral gavage on the alternate days over a two-week period. The combination of CFM-4.16 and Sorafenib provoked a significantly reduced tumor volumes when compared with those noted in the control, placebo-treated animals. Together with our *in vitro* studies demonstrating a robust synergism between CFM-4.16 and Sorafenib, our current data provide us with a further rationale to develop CFM compounds and their formulations for sensitizing parental and resistant NSCLCs to Raf-targeting therapeutics.

## MATERIALS AND METHODS

### Cell culture, reagents and chemicals

Structure and synthesis of CFM-4, -4.16, and -4.17 compounds have been recently described [21, 22]. A stock solution of 10–50 mM of each CFM was prepared in dimethyl sulfoxide (DMSO) and stored at –20° C. 3-[4,5-Dimethylthiazol-2-yl]-2,5diphenyltetrazolium bromide (MTT) were obtained from Sigma-Aldrich, St Louis, MO, USA). The mToR inhibitor Everolimus, PARP inhibitor Olaparib, CDK4/6 inhibitor Palbocicilib, Raf inhibitors Sorafenib and Dabrafenib, Src inhibitor Dasatinib, MET inhibitor Tivatinib, p38 inhibitors Losmapimod and Doramapimod, as well as the EGFR TKIs Gefitinib, Erlotinib, Rociletinib, and Osimertinib were all purchased either from SelleckChem, Boston, MA, USA or ApexBio, Houston, TX, USA. The ERK1/2 inhibitor Ulixertinib was obtained from Chemietek, Indianapolis, IN, USA. Each compound was dissolved in DMSO to obtain a 50 mM stock solution and stored at –20 until needed. We purchased all other analytical reagent grade chemicals from Sigma-Aldrich (St Louis, MO) and used them without further purification.

DMEM, EMEM medium and antibiotics (penicillin and streptomycin) used in this study were purchased from Invitrogen Co. (Carlsbad, CA, USA). Fetal bovine serum (FBS) and DMSO were obtained from Denville Scientific Inc. (Metuchen, NJ, USA), and Fisher Scientific (Fair Lawn, NJ, USA), respectively. The Protein Assay Kit was purchased from Bio-Rad Laboratories (Hercules, CA, USA). The mouse monoclonal antibodies for b–actin were acquired from Sigma-Aldrich (St. Louis, MO, USA). We purchased antibodies for α-tubulin (rabbit polyclonal), Cyclin B1 (V152, mouse monoclonal), Cleaved Caspase-8 (IC12, mouse monoclonal), cleaved PARP (Asp214; mouse monoclonal), RIPK1 (Cat#3493; rabbit monoclonal), phospho (T180/Y182) and total p38α/β, phospho (T183/Y185) and total JNK1/2 SAPKs, total and phospho-STAT3 (Y705), total and phospho-MET (Y1234/1235), total and phospho (Y416)-Src, total and phospho-AKT (S473) and T(308), total and phospho (T389)-p70S6K, total and phospho (S2448)-mToR1, total and phospho-(Y1068) EGFR, total and phospho-(S536) p65/RelA NF-κB subunit, total and phospho (S445) B-Raf from Cell Signaling Technology (Beverly, MA, USA). We have previously described generation and characterization of the anti-CARP-1 rabbit polyclonal antibodies [7].

The human NSCLC H1299, A549, H23, H460, HCC827, and H1975 were obtained from ATCC and validated as described before [43]. The validated TNBC MDA-MB-231, MDA-MB-468, HCC-70, HCC-1806, HCC-1937, and Hs578T were obtained from the Karmanos Cancer Institute Biobanking and Correlative Sciences Core and kindly provided by Dr. Julie Boerner. The RCC A498 cells were from ATCC and kindly provided by Dr. Rajvir Dahiya (UCSF). The HLRCC (UOK 262) cells were kindly provided by Drs. Marsten Lanahan (NCI). All the cells were routinely maintained as described before [38, 39]. All the cell culture media were supplemented with 10% FBS, 100 units/ml of penicillin, and 100 μg/ml of streptomycin, and the cells were kept at 37° C and 5% CO2. For cell growth and MTT studies, the cells were cultured in fresh media with 5% FBS prior to their treatments with various agents.

### Generation of cells resistant everolimus and EGFR TKIs

Isolation and characterization of Gemcitabine-resistant H23 NSCLC cells as well as Everolimus-resistant RCC cells have been described before [22, 43]. The NSCLC HCC827 cells were cultured in continuous presence of escalating doses of Erlotinib starting with 100 nM to eventual dose of 4 μM over a period of 1 year. Multiple, Erlotinib-resistant sublines were isolated and cultured routinely in 2 μM Erlotinib. The H1975 NSCLC cells were separately cultured in continuous presence of escalating doses of Rociletinib or Osimertinib starting with 150nM to eventual dose of 4 μM for each inhibitor over a period of 12 or 6 months, respectively. Multiple, Rociletinib-resistant H1975 cells were isolated and cultured routinely in 2 μM Rociletinib. In the case of Osimertinib-resistant H1975 cells, a pooled population was obtained and cultured routinely in 2 μM Osimertinib. The parental and EGFR TKI-resistant NSCLC cells were characterized for their growth inhibitory (GI_50_) dose of respective TKI by the MTT-based viability assays as below.

### Cell viability assays

The cytotoxicity of CFM-4, -4.16, -4.17, TKIs (Erlotinib, Rocilitinib, Osimertinib, Gefitinib, Dasatinib, Tivatinib), and p38 inhibitors (Losmapimod and Doramapimod) was assessed by MTT assay. First, we seeded 5 × 10^3^ cells in the 96-well plate in triplicate, allowed the cells to grow in fresh culture media for another 24 h, and treated them with respective agents for the noted dose and time. Control cells were treated with 0.1% DMSO in culture medium. After treatment, 20 μL of 1 mg/ml of MTT was added to each well and cells were incubated for 2–4 h at 37° C. MTT was removed, and the resulting formazan products were dissolved by adding 50μl DMSO/well. The colorimetric analysis was carried out using a multi-label plate reader at 570 nm (Victor3; PerkinElmer, Wellesley, MA).

### Combined drug effect analysis

The median-effect principle of Chou and Talalay was the basis for conducting the CFM-4.16 effect analysis in combination with various targeted therapeutics [44]. The combination index (CI) values were calculated for determining the mode of interaction (synergism, antagonism and additive effect) between CFM-4.16 and therapeutics such as Sorafenib (targets B-Raf), Dabrafenib (targets B-Raf), Everolimus (Targets mToR1), Olaparib (targets PARP), or Palbociclib (targets CDK4/6) as described by Chou and Talalay [45, 46]. We applied CalcuSyn software version 2 (Biosoft) for above drug combination analysis essentially as described previously [47] and following software guidelines. The CI values presented in Table 2 are based on the effective dose (ED)50 of each compound for the indicated cell line as suggested by the CalcuSyn software.

### Generation of CARP-1 knock-down NSCLC cells

The HCC827 NSCLC parental cells were transfected with vector plasmid pcDNA3/hygro or plasmid expressing CARP-1 anti-sense (Clone 1.6, ref 7). Multiple, stable sublines for hygromycin resistance were selected in the presence of 400 mg/ml hygromycin (#10687010, InVitrogen Inc) following methods described before [7]. Knock-down of CARP-1 in multiple, CARP-1 anti-sense-expressing sublines was determined by western blot analysis of the cell lysates as detailed below. We then determined viabilities of the parental, and vector or CARP-1 antisense plasmid-transfected HCC 827 cells in the presence of CFM-4.16 or Erlotinib by above described MTT assays.

### Western blot analysis

For protein expression analysis, we conducted western blot experiments. The NSCLC cells were treated with 0.1% DMSO/Vehicle (Control) or indicated dose and time of the noted compound, and were lysed to prepare protein extracts. Cells were harvested and lysed in RIPA buffer (50 mM Tris-HCI, pH 8.0, 150 mM sodium chloride, 1.0% NP-40, 0.5% sodium deoxycholate, 0.1% sodium dodecyl sulfate (SDS), and 0.1% of protease inhibitor cocktail) for 20 min at 4° C. The lysates were then centrifuged at 14,000 rpm at 4° C for 15 min to get rid of debris. We then determined the protein concentrations of whole cell lysates using the Protein Assay Kit. Supernatant proteins, 50 μg from each sample, were separated by SDS-10% polyacrylamide gel electrophoresis (SDS-PAGE) and transferred to polyvinylidene difluoride (PVDF) membrane (Bio-Rad, Hercules, CA, USA) by standard procedures. The membranes were hybridized with primary antibodies followed by incubation with appropriate secondary antibodies. The antibody-bound proteins were visualized by treatment with the chemiluminescence detection reagent (Amersham Biosciences) according to the manufacturer’s instructions, followed by exposure to X-ray film (Kodak X-Omat). The same membranes were then re-probed with either the anti-β actin or anti-α tubulin antibody, which was used as an internal control for protein loading.

### Preparation and characterization of CFM-4.16 NLF

The procedure to prepare CFM-4.16 NLF was essentially the same as described in earlier communications [21]. Briefly, appropriate amount of CFM-4.16 was blended with Compritol 888ATO, Miglyol 812N, and Geleol, and the mixture was melted at 70° C to form a uniform and clear oil phase. To this, aqueous phase consisting of dispersing surfactant Tween 80 and Vitamin E TPGS in double distilled water was added drop wise to the oil phase at 70° C. The coarse emulsion was then homogenized for 15 min under high pressure using NanoDebee for about 5 cycles. The formulation was then characterized for its particle size and zetapotential and drug release using methods described elsewhere [20, 21].

### Cell migration and clonogenic assays

The NSCLC cells migration in the absence or presence of CFMs was measured by the “scratch/wound healing” assay essentially as described before [20–22]. Briefly, cells were seeded in a 6-well plate (∼10,000 cells/well), and when attached, a scratch was created in the cell monolayer using sterile pipette tip. The cells were then allowed to continue growing in the absence (Control) or presence of noted dose of each of the agents for indicated time periods. The cells were photographed at the beginning and at regular intervals during the treatment period, and the images from control cells were compared with the treated cells to determine the migration of the cells. The photomicrographs of the cells were recorded under different magnifications utilizing Zeiss microscope with attached 35 mm camera.

Clonogenic assay: A soft-agar sandwitch assay was performed. Cells were sandwiched between 0.6% and 0.3% agarose in DMEM medium containing 5% FBS in a six-well chamber (500 cells/chamber), and treated with buffer (Control), or respective agent for noted time and dose at 37° C humidified CO_2_ incubator. The colonies from multiple random fields were counted, compared to control and photographed essentially as described before [20–22].

### Three-dimensional NSCLC sphere assays

The parental and TKI-resistant NSCLC cells from a two-dimensional culture plate with ∼70–80% confluence were utilized for 3D assays. We performed the three-dimensional NSCLC sphere cultures by essentially following the methods described by us before [22, 22]. Briefly, the cells were washed twice in 1 × PBS and trypsinized following established protocols. We then pelleted the cells at 200 × g at room temperature, and re-suspended them in 5 ml of sphere media (DMEM/F12 supplemented with 2 mM L-glutamine, 100 U/ml penicillin, 100 U/ml streptomycin, 1 × B27 supplement, 20 ng/ml recombinant human epidermal growth factor (EGF; Sigma), and 10 ng/ml recombinant human basic fibroblast growth factor (bFGF; R&D Systems). We seeded ∼5000 viable cells per ml in an ultra-low adherent 60 mm plate and incubated them at 37° C and 5% CO_2_ for two weeks without disturbing the plates. After the spheres formed, we added fresh media with or without 10 µM CFM-4.16 and continued incubating cells for additional 24 h at 37° C and 5% CO_2_. At the end of the incubation period, we photographed the spheres in the untreated and treated plates as described [48].

### Establishment of NSCLC cell-derived xenografts in immunocompromised mice

The experiments involving generation of Rociletinib-resistant H1975 NSCLC cell-derived sub-cutaneous xenografts were performed according to our previously published methods and protocols approved by the Institutional Laboratory Animal care & Use Committees at the Florida A&M University [20, 21]. Female, 4–6-weeks old (20–25 g) Balb/c nude mice were purchased from Charles River Laboratories (Horsham, PA, USA). Following suitable acclimation of animals, Five million H1975 (rocelitinib-resistant) cells were suspended in Hanks Balanced salt solution and implanted s.c. in right flanks of the nude mice using a 27-gauge needle. Tumors were allowed to grow for 15 days and when tumors became palpable (200 mm3), the mice were randomly assigned to treatment or control groups with 6 animals in each group. The treatment groups consisted of control, CFM-4.16 NLF (50 mg/kg), Sorafenib (30 mg/kg), and CFM-4.16 NLF plus Sorafenib every alternate day for 14 days. All preparations were given by oral gavage. The mice were followed for their tumor burden and mobility for the next three weeks after which the experiment was terminated and the mice were sacrificed. Tumor tissues were collected immediately after tumor volume measurement. Tumor volume was calculated by the modified ellipsoidal formula: Tumor volume = 1/2(length × width2). Representative tumor samples were stored at –80° C and in 10% formalin for subsequent analysis.

### Statistical analysis

The statistical analysis was done using Prism 6.0 software (Graph Pad Software Inc., San Diego, CA, USA). The data were expressed as mean ± SEM and analyzed using a two-tailed Student *t*-test or one-way ANOVA followed by a post hoc test. A *p* value of < 0.05 was considered statistically significant.

## SUPPLEMENTARY MATERIALS FIGURES


