# Supplementary material for: A CARP-1 functional mimetic compound is synergistic with BRAF-targeting in non-small cell lung cancers
Source: Oncotarget. 2018 Jul 3;9(51):29680–97. doi: 10.18632/oncotarget.25671 (PMC6049854; doi:10.18632/oncotarget.25671)
Supplement: Supplementary file 1 [file oncotarget-09-29680-s001.pdf]

A CARP-1 functional mimetic compound is synergistic with braf-targeting in non-small cell lung cancers

SUPPLEMENTARY MATERIALS

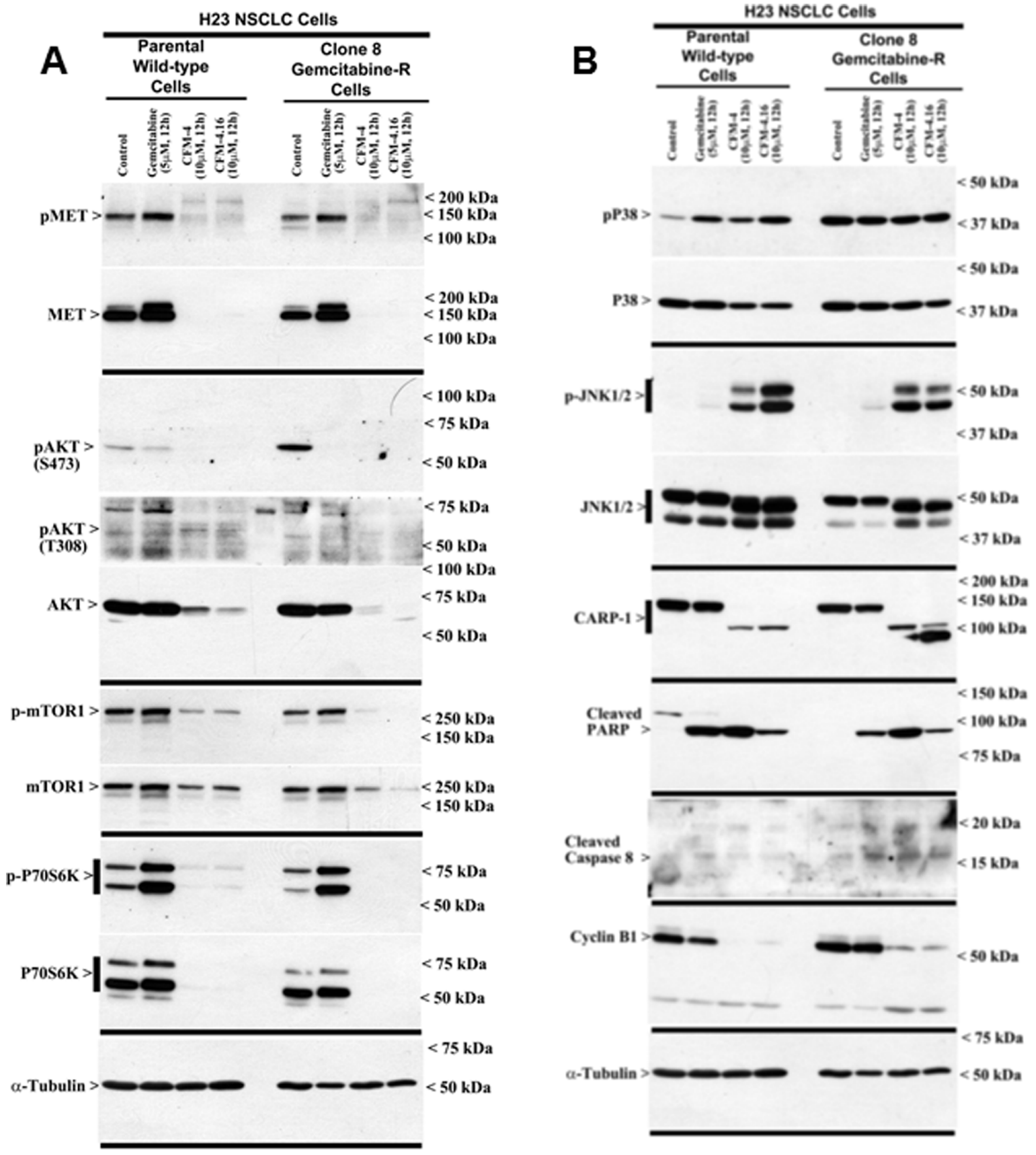

Supplementary Figure 1: CFM-4.16 inhibits expression and activation of oncogenes (**A**) and stimulates apoptosis (**B**) in parental and Gemcitabine-resistant NSCLC cells. Indicated parental and Gemcitabine-resistant NSCLC cells were either untreated (Control), treated with Gemcitabine, CFM-4, or CFM-4.16 for noted dose and time. Cell lysates were analyzed by Western blotting (WB) as in Methods for levels of Oncogenes MET, AKT, mToR1, p70S6K in panel A while expression of growth inhibitory and proapoptotic genes such as CARP-1, cyclin B1, cleaved PARP and caspase-8, and activation (phosphorylation) of pro-apoptotic p38 and JNK1/2 SAPKs was analyzed in panel B. The western blot membranes were subsequently probed with α-tubulin antibodies to assess equal loading. The presence of respective protein is indicated by an arrowhead on the left side of each blot. Approximate location of various molecular weight markers is indicated on the right side of each blot. kDa, kilodalton.

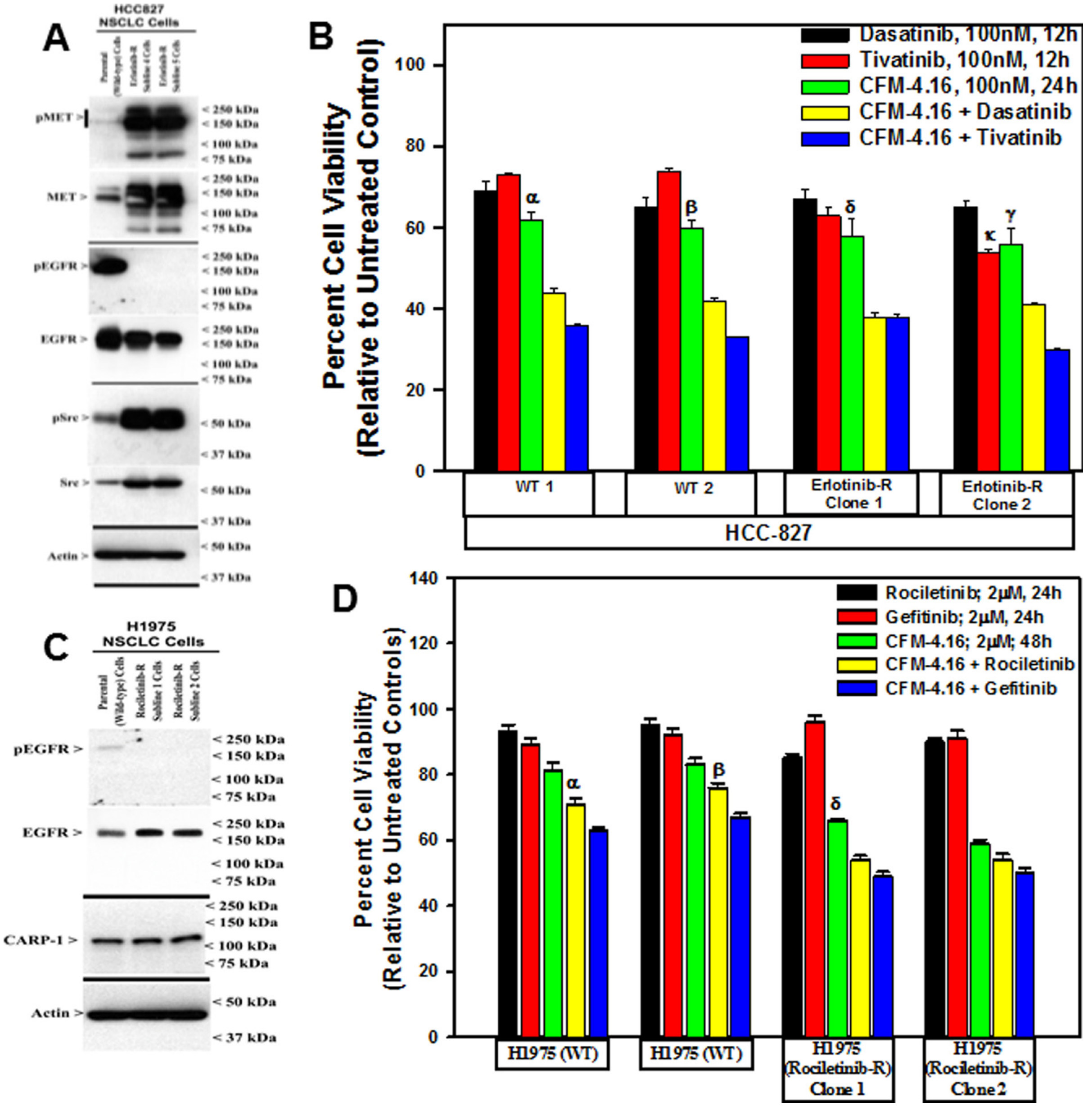

Supplementary Figure 2: TKI-resistant NSCLC cells have elevated expression and activation of oncogenes (**A**, **C**), and CFM-4.16 has superior efficacy when used in combination with therapeutics that target MET, Src (**B**), or EGFR (**D**). Lysates derived from indicated parental and Erlotinib-resistant NSCLC cells were analyzed by Western blotting (WB) as in Methods for levels and activities (phosphorylation) of Oncogenes MET, AKT, mToR1, p70S6K in (A) while lysates derived from parental and Rociletinib-resistant NSCLC cells were analyzed for expression and activation (phosphorylation) of EGFR in (C). The WB membranes were subsequently probed with α-tubulin antibodies to assess equal loading. The presence of respective protein is indicated by an arrowhead on the left side of each blot. Approximate location of various molecular weight markers is indicated on the right side of each blot. kDa, kilodalton. (B, C) Noted cell lines were either treated with DMSO (Control) or with various compounds for indicated dose and time. Cell viability was determined by MTT assay. The data in the histograms represent means of three independent experiments; bars, S.E. α, β, δ, κ, γ, ρ, $p = < 0.05$ relative to the respective DMSO-treated controls.

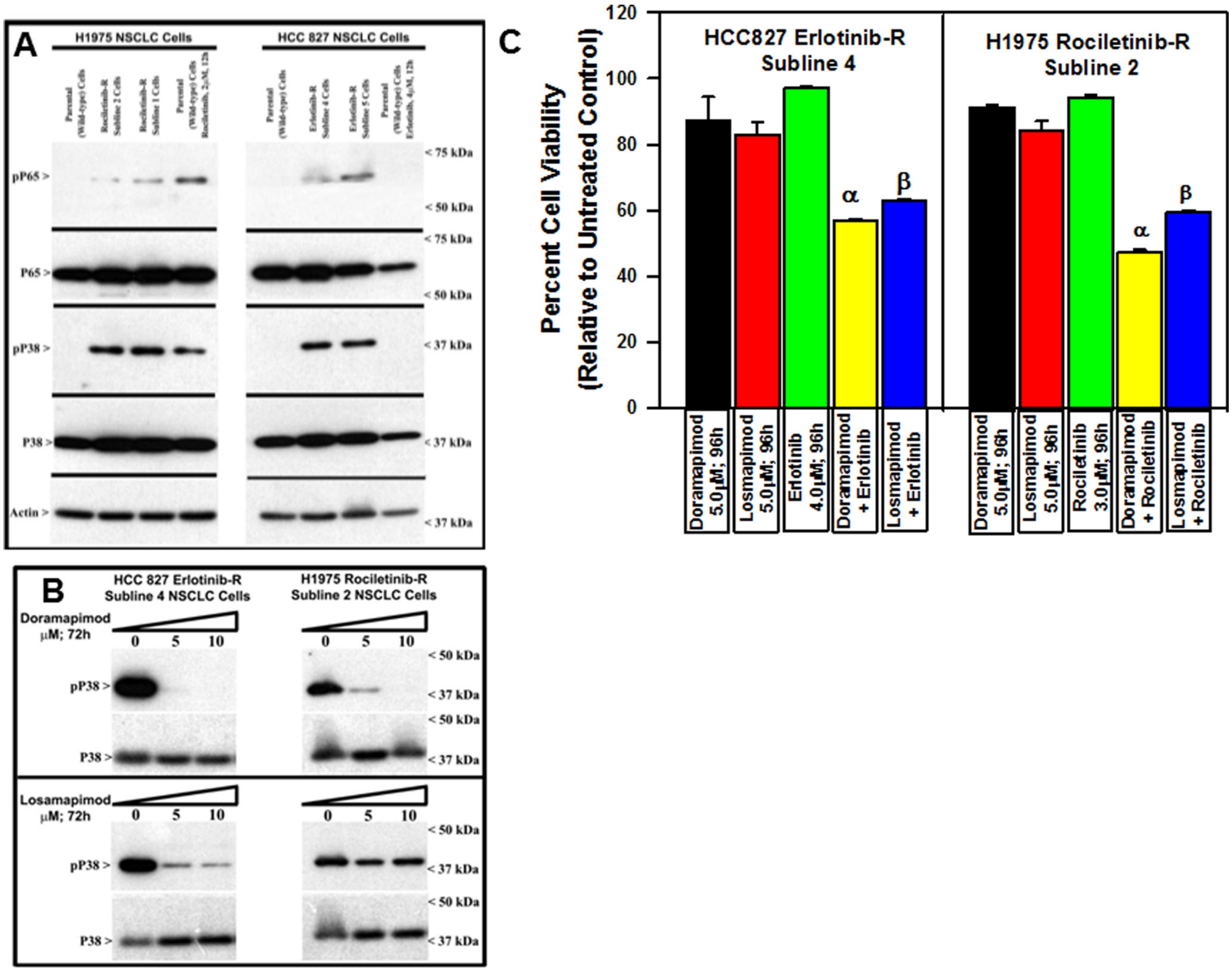

Supplementary Figure 3: Aberrant Gene Expression in TKI-resistant NSCLC cells. (**A**) WB showing expression of NF-κB subunit p65 (total and phosphorylated) and p38 MAPK/SAPK (total and phosphorylated) in the indicated parental and TKI-resistant NSCLC cells. (**B**) WB showing expression of phosphorylated and total p38 proteins in indicated, TKI-R sublines treated with noted doses of p38 inhibitors. The numbers on the right of each autorad in A and B indicate migration of protein molecular weight markers (Bio-Rad). (**C**) The Erlotinib-R subline 4 and Rociletinib-R subline 2 were treated with DMSO (Control) or with noted agents for indicated dose and time. Cell viability was determined by MTT assay. The histogram columns indicate live/viable cells and represent means of two independent experiments; bars, S.E. α, β, *p* = < 0.05 relative to the respective DMSO-treated controls. Of note is that all the TKI-resistant sublines of Table 1 had elevated levels of p-p38, and p-p65 (not shown). For brevity, (A) shows data from two, TKI-resistant HCC827 and H1975 cells.

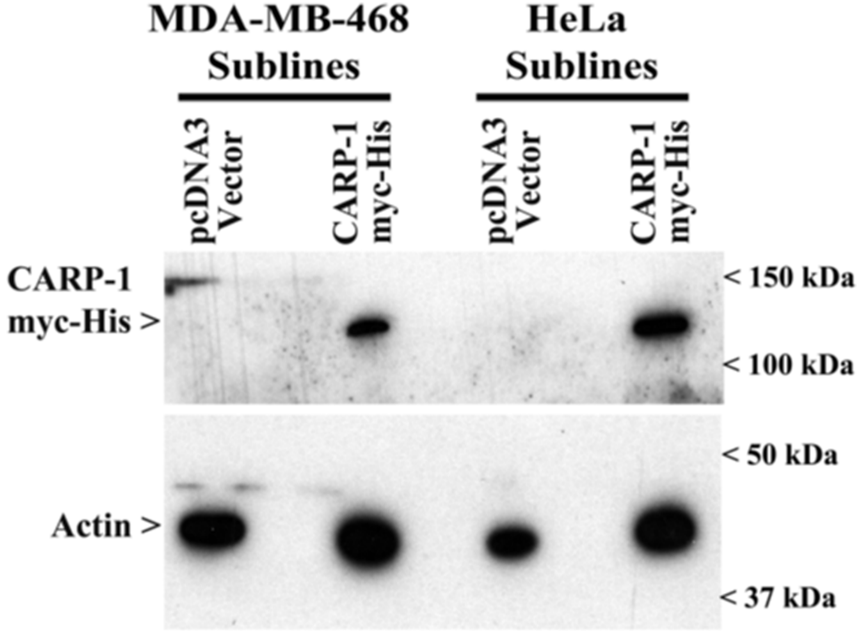

Supplementary Figure 4: Stable expression of CARP-1-myc-His. Indicated cell lines were separately transfected with pcDNA3 Vector or pcDNA3-CARP-1-myc-His plasmids, and sublines expressing respective plasmid were selected in the chronic presence of neomycin (800 ug/ml) over a period of 12–16 weeks. Cell lysates from individual subline were analyzed by WB using anti-myc-tag antibodies (upper blot) or anti-actin antibodies (lower blot). The numbers on the right of each autorad indicate migration of protein molecular weight markers (Bio-Rad).

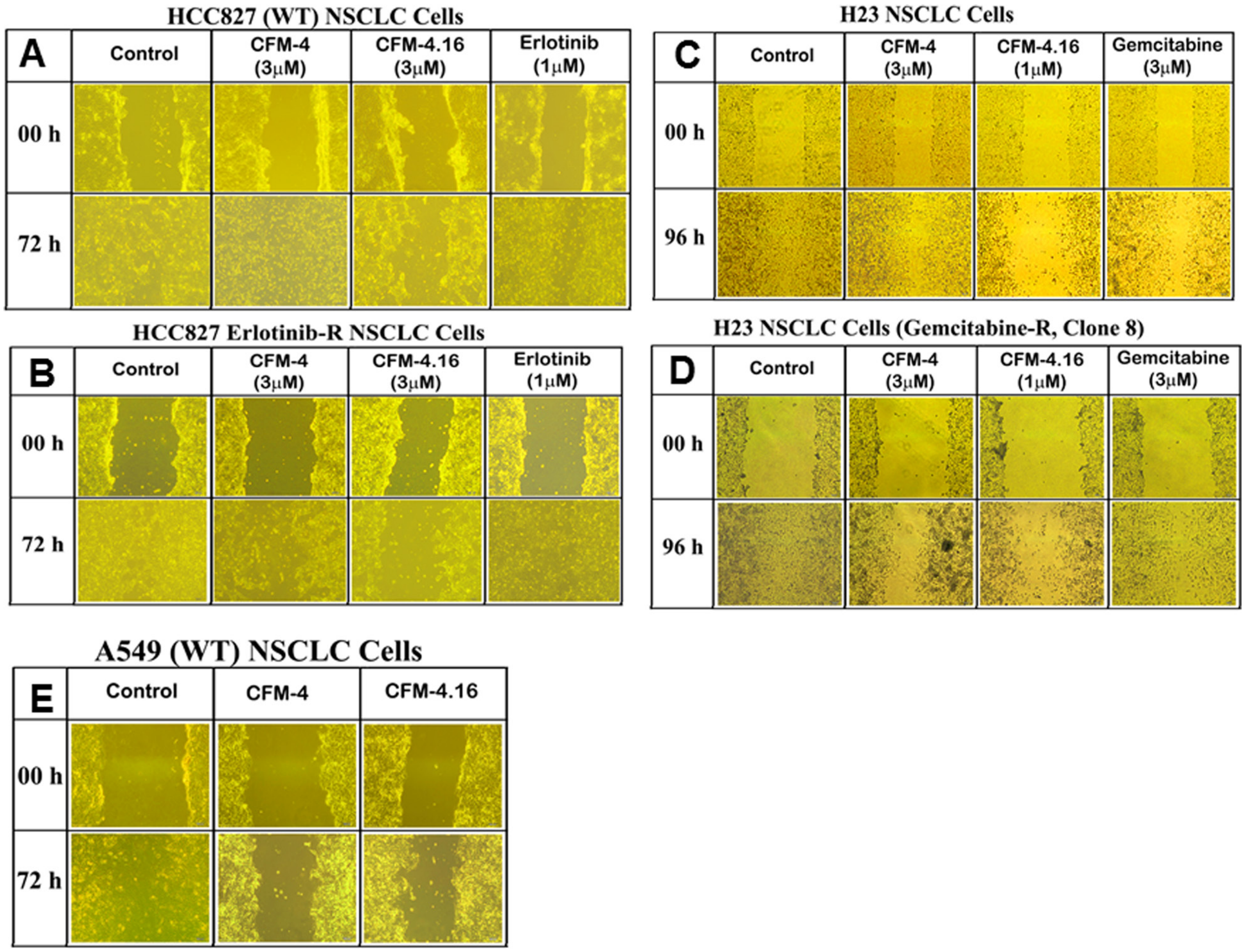

Supplementary Figure 5: CFM-4.16 inhibits motility of the parental and TKI-resistant NSCLC cells. (**A**–**E**), Indicated NSCLC cells were either untreated (Control), treated with 3 µM of respective CFMs, 3.0 µM of Gemcitabine, or 1.0 µM of Erlotinib for 72 h, and subjected to scratch assays as described in Methods. The cells growth in the scratch assay was recorded by photography as detailed in Methods. Representative photomicrographs of untreated and treated NSCLC cells are shown.

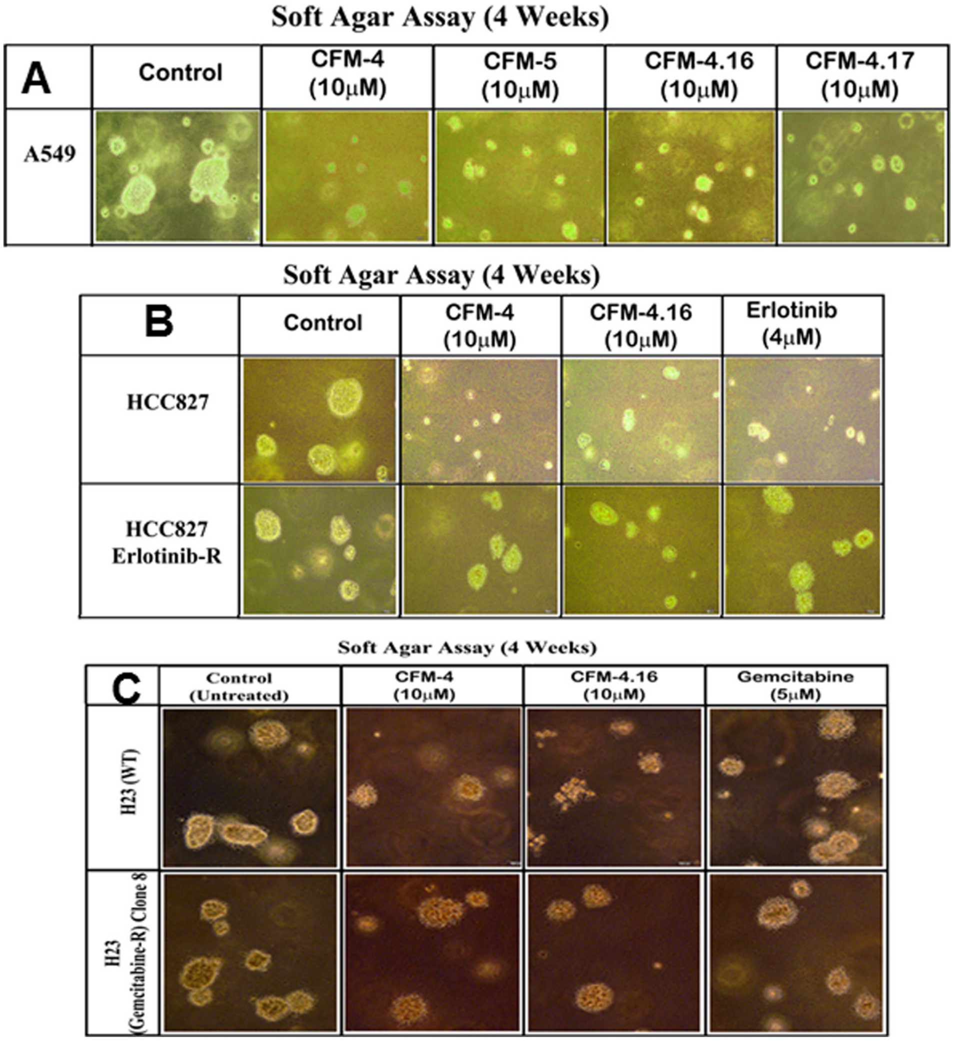

Supplementary Figure 6: CFM-4.16 inhibits growth of the parental and TKI-resistant NSCLC cells in soft agar. (A–C) Indicated NSCLC cells were seeded in soft agar and either untreated (Control), treated with noted doses of CFM-4, CFM-4.16, Erlotinib, or Gemcitabine for 4 weeks. The number of colonies of cells were recorded by photography as detailed in Methods. Representative photomicrographs of untreated and treated NSCLC cells are shown.
